# Toward minimal SNP sets for record-matching with CODIS STR profiles

**DOI:** 10.1038/s41431-025-01941-7

**Published:** 2025-09-22

**Authors:** Tamara Gjorgjieva, Noah A. Rosenberg

**Affiliations:** 1 Department of Genetics, Stanford University, Stanford, CA USA; 2 Department of Biology, Stanford University, Stanford, CA USA

**Keywords:** Population genetics, Genetic markers

## Abstract

Genetic record-matching is a technique by which profiles with one set of genetic markers can be queried against databases of profiles with a different set of markers to determine if profiles containing different marker sets trace to the same individual. In forensic genetics, the potential for using genetic record-matching to test single-nucleotide polymorphism (SNP) profiles for genetic matches to short-tandem repeat (STR) profiles could enable development of backward-compatible SNP marker systems to ultimately replace existing forensic STR systems. This study aims to identify minimal SNP sets for achieving record-matching accuracies comparable to those previously observed with tens or hundreds of thousands of SNPs. Using phased SNP–STR reference data in a worldwide panel of individuals, we evaluate record-matching accuracy with SNP sets chosen by each of a variety of SNP selection strategies. When selecting SNPs randomly, ~9000 SNPs are required for achieving record-matching accuracy comparable to that seen with the full SNP set in the “needle-in-haystack” matching scenario, namely 99% of SNP and STR profiles correctly paired with no false-positive identifications in the median accuracy for test sets of size 626 profile pairs. Selecting SNPs based on various thresholds for their minimal minor allele frequency and physical distance to the STR, however, panels of 1800 SNPs, and as few as 900 SNPs, suffice. These results advance toward a potential minimal size for backward-compatible forensic SNP systems that proceed by genetic record-matching.

## Introduction

Short tandem repeats (STRs) are genomic regions that contain short repeated sequences, with the number of repeats varying across individuals. Because STRs often possess considerable inter-individual variability, and because they became widely available as forensic genetic systems were developed in the 1990s, they have been the basis for genetic marker systems routinely used for individual identification in forensic genetics. Millions of profiles have now been obtained with sets of ~13-20 STRs for use in forensic genetic identification.

Single-nucleotide polymorphisms (SNPs), variable genomic sites that typically possess two distinct allelic types at a specific genomic location, offer potential advantages over STRs in a forensic context [1–3]. SNPs are genomically abundant and suited to high-throughput analysis with little genotyping error. Their uses elsewhere in human genetics have generated significant advances in genotyping technology and statistical methods. Notably, SNPs can be more reliably typed from degraded samples with limited quantities of DNA than can STRs, potentially opening new possibilities for forensic analysis in an expanded set of scenarios.

Studies have long been developing SNP panels for diverse forensic genetics tasks [4–7]. For the central task of individual identification, a recent development is the introduction of a 5422-SNP panel that contains SNPs chosen to be informative in various forensic dimensions and that has been implemented for identifying unidentified human remains [8]. A SNP panel of this type—with potential for standardization, genotyping with small DNA quantities, and reduced false positive rates in scenarios such as assessment if a profile is included in a DNA mixture—could eventually replace STR panels for routine forensic use. However, a challenge in the path to replacing STR panels by SNPs is that profiles obtained with one set of loci cannot be easily searched for genetic matches to profiles obtained on another, disjoint set.

To assist with solving this problem of “backward compatibility” between SNP and STR loci, the genetic record-matching technique uses correlation patterns between STRs and genomically adjacent SNPs to statistically assess if profiles obtained from non-overlapping SNP and STR marker sets belong to the same individual [9] or to close relatives [10]. In recent computations with genetic record-matching, Kim & Rosenberg [11] have estimated that profiles belonging to the same individual can be successfully matched between two databases containing 2504 individuals sampled from diverse worldwide populations: an STR database with 15 loci and a SNP database with 161,968 loci in the 1-Mb neighborhoods of the STRs. Kim & Rosenberg [11] also saw that if the number of loci is reduced to 8000–16,000 (5–10% genomic coverage), record-matching accuracy is comparable to that achieved with the larger set.

What is the smallest SNP panel that can enable backward-compatible record-matching of SNP profiles with STR profiles? This study seeks to identify minimal sets of SNPs for record-matching. Whereas Kim & Rosenberg [11] selected SNPs at random in neighborhoods of the STRs that appear in STR profiles, we select SNPs based on various specified characteristics. We evaluate the performance of the resulting SNP panels by computing their record-matching accuracies in the same set of individuals studied by Kim & Rosenberg [11]. We find that certain sets with as few as 900 SNPs surrounding 18 STRs achieve comparable record-matching accuracy to the 8000–16,000 SNPs surrounding 15 CODIS STRs in [11]. The results indicate that considerable scope exists for optimizing SNP panels for backward-compatible genetic record-matching with STR panels.

## Materials and methods

We follow [11] with differences as noted below. Kim & Rosenberg [11] considered 2504 individuals, examining 161,968 SNPs located in 1-Mb regions surrounding 15 of the CODIS STRs. They evaluated the record-matching accuracy based on the full SNP set, as well as with randomly selected fractions of the SNPs. We use the same set of individuals and the same record-matching pipeline. However, we use 18 rather than 15 CODIS STRs, with 192,672 total SNPs in the 1-Mb windows. We consider many strategies for SNP selection.

### Dataset

Kim & Rosenberg [11] started from the phased reference SNP–STR panel derived from the 1000 Genomes Project phase 3 data [12]. The dataset considers 2504 individuals in 26 worldwide subpopulations. Saini et al. [12] used family pedigrees to develop a genome-wide data set containing phased haplotypes of STRs and SNPs.

Kim & Rosenberg [11] had considered 15 CODIS STRs that were available in both the 1000 Genomes Project and the Human Genome Diversity Panel. Here, we consider only the 1000 Genomes, and we analyze 18 CODIS STRs: 11 of the 13 original CODIS STRs (CSF1PO, D13S317, D18S51, D3S1358, D5S818, D7S820, D8S1179, FGA, TH01, TPOX, and vWA) and the 7 loci added in 2017 (D1S1656, D2S441, D2S1338, D10S1248, D12S391, D19S433, and D22S1045) [13]. For each STR, we examine all SNPs in a 1-Mb window (i.e. distance<500 kb from a point regarded as the STR location), an average of ~11,000 SNPs per STR (Table S1).

In some analyses, we use the 1000 Genomes [14] assignments of populations to five continental super-populations—Africa (AFR), the Americas (AMR), East Asia (EAS), Europe (EUR), and South Asia (SAS). With the 1000 Genomes abbreviations, population assignments are ESN, GWD, LWK, MSL, YRI to AFR; ACB, ASW, CLM, MXL, PEL, PUR to AMR; CDX, CHB, CHD, CHS, JPT, KHV to EAS; CEPH, FIN, GBR, IBS, TSI to EUR; and BEB, GIH, ITU, PJL, STU to SAS.

### Record-matching

Genetic record-matching uses linkage disequilibrium (LD) between SNPs and STRs to identify matches between two profiles with non-overlapping sets of loci, in this case forensic STR profiles and SNP profiles. A match suggests that two profiles trace to the same individual.

The steps in testing record-matching methods include: (a) partitioning individuals into training and test sets, (b) using the phased haplotypes in the training set to assist in performing imputation of STRs from SNP profiles in the test set, (c) matching STR profiles to SNP profiles, and (d) computing record-matching accuracies from match-score matrices (Fig. S1A). For the statistical framework of genetic record-matching, see the Methods of [11].

### Training and test sets of individuals

Following [9–11], we partitioned the data into disjoint training and test sets, with 75% of individuals in the training set. We generated 10 replicate partitions and used them in subsequent experiments.

### Imputation with BEAGLE

The training set included individuals with phased SNP–STR haplotypes, whereas the test set contained individuals with only SNPs, with STRs kept hidden. To estimate the unobserved STR genotype probabilities in the test set, we used BEAGLE [15] to impute STR genotypes from SNP profiles, relying on the phased SNP–STR haplotype reference panel from the training set. We followed BEAGLE settings of Kim & Rosenberg [11].

### Matching SNP and STR profiles

We computed pairwise match scores to populate a match-score matrix ***M*** with size ***N×N***, where ***N*** is the total number of individuals in the test dataset. Following Edge & Rosenberg [9], match scores correspond to log-likelihood ratios comparing the hypotheses that two profiles do or do not belong to the same individual. In the match-score matrix, rows correspond to individuals’ SNP profiles, and columns to their STR profiles. Based on this matrix, we then assigned matches under four matching scenarios.

In one-to-one matching, for a given row or column of ***M***, exactly one pair is selected as a match; we proceed by the Hungarian algorithm [16]. In one-to-many matching with a query SNP profile (“SNP query”), the STR profile with the highest match score among STR profiles is assigned. An STR profile can have the highest match score for multiple query SNP profiles. Similarly, in one-to-many matching with a query STR profile (“STR query”), the SNP profile with the highest match score among SNP profiles is assigned. A SNP profile can have the highest match score for multiple query STR profiles. In needle-in-haystack matching, designed to mimic a database query with a specific profile, a match is identified between a SNP profile and STR profile if the match score exceeds that of all non-matches.

Following Edge & Rosenberg [9] and Kim et al. [10], these scenarios represent progressively more challenging cases. One-to-one matching assumes each SNP profile has exactly one STR profile match and vice versa. One-to-many matching introduces the possibility of errors in which multiple profiles of one type are mistakenly assigned to the same profile of the other type. In needle-in-haystack matching, a proposed match must not only be the best such proposal for a query profile, it must also exceed a high threshold for the match score.

### Computing record-matching accuracies

In one-to-one and one-to-many matching, the accuracy of record-matching is defined as the fraction of pairs that are correctly matched among ***N*** true matches. In needle-in-haystack matching, the accuracy is the proportion of true matches that have higher match scores than the largest match score across all non-matching pairs.

### SNP selection

Our deliberate SNP selection prior to record-matching extends beyond the random SNP selection of [11]. We perform record-matching (Fig. S1A) using SNP panels produced by random sampling among (a) all SNPs in 1-Mb regions around CODIS STRs, (b) SNPs that lie in 1-Mb windows and that possess a specific characteristic, and (c) SNPs that lie in 1-Mb windows that possess two or more specific characteristics (Fig. S1B).

### Random sampling among all SNPs

For our initial experiments, we randomly selected 10, 25, 50, 75, 100, 125, 250, 500, 100, 5000, and all SNPs per STR, resulting in SNP sets of size of 180, 450, 900, 1350, 1800, 2250, 4500, 9000, 18,000, 90,000, and 192,672. We then used these SNP sets to perform record-matching in 100 replicates. For sizes 180, 450, …, 90,000, we generated 10 random samples of SNPs and considered each in the 10 replicate partitions of individuals into training and test sets. For size 192,672, we simply used 100 partitions (including the 10 used for other sizes). Note that Kim & Rosenberg [11] described SNP selection in terms of fractions of the SNPs (e.g., 1% of SNPs); here we instead refer to SNP counts.

### Random sampling among SNPs that possess a specific characteristic

For our next experiments, we restricted SNP sets to SNPs that possessed a specific characteristic. Statistics for identifying SNPs included: minor allele frequency in the full data of 2504 individuals (MAF), minimal MAF across the 5 super-populations (hereafter “pop-MAF”), distance to the CODIS STR in base pairs, and *D*’_avg_, a measure of LD of a SNP with the CODIS STR (described below).

For each statistic, we considered SNPs satisfying each of three conditions. Among SNPs satisfying a condition, we randomly selected 25, 50, 75, and 100 SNPs per CODIS STR, for a total set of size 450, 900, 1350, and 1800. We then performed record-matching and evaluated the accuracy in 100 replicates (10 partitions with each of 10 random SNP sets).

Conditions on the statistics were as follows:

1. *MAF*. We considered three MAF conditions: SNPs with MAF ≥ 1%, MAF ≥ 5%, and MAF ≥ 10%.
2. *Pop-MAF*. Using the MAF values in each of the five super-populations, we considered three pop-MAF conditions: pop-MAF > 0%, pop-MAF ≥ 1%, and pop-MAF ≥ 5%. Note that pop-MAF > 0% is equivalent to selecting SNPs variable in each super-population.
3. *Distance to CODIS STR*. We considered three conditions: SNPs with distance less than or equal to 0.25 Mb, 0.125 Mb, or 0.0625 Mb to the CODIS STR.
4. *D’*_*avg*_ *with CODIS STR*. We considered conditions on the minimal LD between a SNP and the CODIS STR. Payseur et al. [17] defined an LD statistic for multiallelic loci:
  $${D}_{{avg}}^{{\prime} }={\sum }_{i=1}^{k}{\sum }_{j=1}^{l}{p}_{i}{q}_{j}|{D}_{{ij}}^{{\prime} }|.$$
  Here, *k* refers to the number of alleles at the first locus (say, the STR), *l* to that of the second locus (the SNP), *p*_i_ to the frequency of STR allele *i*, and *q*_j_ to that of SNP allele *j*. |*D*’_ij_| is the standard |*D*’| statistic for biallelic loci, where presence or absence is tabulated for allele *i* at the STR and allele *j* at the SNP. Using phased CODIS SNP–STR haplotypes, we computed *D*’_avg_ for each SNP with the STR. We considered three conditions: *D*’_avg_ ≥ 0.3, *D*’_avg_ ≥ 0.5, and *D*’_avg_ ≥ 0.7.

### Random sampling among SNPs that possess two or more specific characteristics

Finally, we considered pairs of conditions consisting of a minimal MAF or pop-MAF and a maximal distance. We applied the MAF and pop-MAF conditions with each distance condition.

With each of the 18 combinations of a MAF or pop-MAF condition and a distance condition, at least 100 SNPs are retained per STR (Table S2A). For each combination, we randomly selected 10, 20, …, 100 SNPs per STR, for total SNP panel sizes of 180, 360, …, 1800. We performed record-matching with these sizes in 100 replicates (10 partitions, 10 SNP sets).

Although the *D*’_avg_ conditions did not perform well individually (see “Results”), we considered them together with the 18 combinations of a condition on MAF or pop-MAF and a distance condition, to produce 54 combinations. We also considered each *D*’_avg_ condition together with the 6 conditions on MAF or pop-MAF, without a distance condition, for 18 more combinations. Finally, we considered each *D*’_avg_ condition together with the 3 distance conditions, for 9 more combinations. Among the 54 + 18 + 9 = 81 combinations, 16 retained at least 100 SNPs per STR (Table S2B). For each of those 16 combinations, we randomly selected 10, 20, …, 100 SNPs per STR, for total SNP panel sizes 180, 360, …, 1800. For each of the 16 combinations and each panel size, we performed record-matching in 100 replicates (10 partitions, 10 SNP sets).

## Results

### All SNPs

**Fig. 1:**
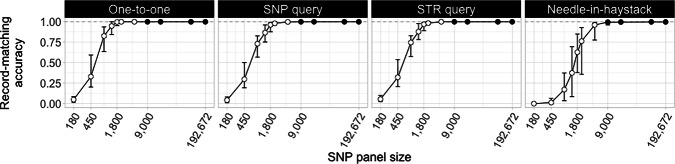
Record-matching accuracies for sets of randomly selected SNPs. The four plots correspond to four different matching scenarios. Each plot shows record-matching accuracies in relation to the total number of SNPs. Points represent the median, and error bars represent the range of record-matching accuracies across 100 replicates; for SNP sets of sizes 180 to 90,000, the 100 replicates correspond to 10 partitions of the individuals and 10 sets of random SNPs. For the set of all 192,672 SNPs, the 100 replicates correspond to 100 partitions. Closed symbols correspond to SNP sets that achieve comparable record-matching accuracies to those obtained with the full set of 192,672 SNPs. The full numerical values are shown in Table S3.

To establish the baseline record-matching accuracy relying on the maximal information available in the study, we first used all 192,672 SNPs in 1-Mb windows around 18 CODIS STRs. Figure 1 plots median record-matching accuracies using the full SNP set, along with ranges across the 100 partitions. Median accuracies are 1 for one-to-one matching (range [1–1]), 1 for SNP query (range [0.998–1]), 1 for STR query (range [1–1]), and 0.998 for needle-in-haystack matching (range [0.987–1]). Numerical values from Fig. 1 appear in Table S3.

Based on these findings, in subsequent analyses, we consider SNP sets with median accuracy 1 for the one-to-one, SNP query, and STR query scenarios and ≥0.99 for the needle-in-haystack scenario to have “comparable accuracy to the full set of SNPs.”

### Randomly sampled SNPs

We next examined random SNP sets. Figure 1 shows the median and range of the record-matching accuracy under the four matching scenarios for random SNP sets of 11 sizes. The figure and associated Table S3 show that random SNP sets of size 9000 or greater produce comparable accuracy to the full set of SNPs.

We conclude from this analysis that accurate record-matching between our SNP and STR databases requires at least 9000 randomly selected SNPs for 18 CODIS STRs (Table 1A). This number corresponds to a fraction ~4.7% of the full set of SNPs, so that our results are consistent with Kim & Rosenberg [11], who found that a genomic coverage of 5–10% suffices to produce record-matching results comparable to those obtained from the full set of SNPs.

**Table 1:** SNP sets for achieving comparable record-matching accuracy to the full data.

			One-to-one		SNP query		STR query		Needle-in-haystack	
	Condition	SNP panel size	Median	Range	Median	Range	Median	Range	Median	Range
**A. Baseline – randomly selected SNPs**										
	Random selection	9000	1	[1–1]	1	[0.997–1]	1	[0.998–1]	0.995	[0.962–1]
**B. Locus characteristics**										
	Pop-MAF ≥ 1%	1800	1	[1–1]	1	[0.997–1]	1	[0.998–1]	0.992	[0.784–1]
	Pop-MAF ≥ 5%	1800	1	[1–1]	1	[0.997–1]	1	[0.998–1]	0.992	[0.896–1]
**C. Combinations of locus characteristics**										
	MAF ≥ 5%, Distance ≤ 0.125 Mb	900	1	[1–1]	1	[0.998–1]	1	[0.997–1]	0.990	[0.879–1]
	MAF ≥ 10%, Distance ≤ 0.125 Mb	900	1	[1–1]	1	[0.998–1]	1	[0.998–1]	0.992	[0.866–1]
	Pop-MAF > 0%, Distance ≤ 0.0625 Mb	900	1	[1–1]	1	[0.998–1]	1	[0.998–1]	0.990	[0.939–1]
	Pop-MAF > 0%, Distance ≤ 0.125 Mb	900	1	[1–1]	1	[0.998–1]	1	[0.997–1]	0.990	[0.799–1]
	Pop-MAF ≥ 1%, Distance ≤ 0.0625 Mb	900	1	[1–1]	1	[0.997–1]	1	[0.998–1]	0.992	[0.936–1]
	Pop-MAF ≥ 5%, Distance ≤ 0.125 Mb	900	1	[1–1]	1	[0.998–1]	1	[0.998–1]	0.994	[0.949–1]

(A) Randomly selected SNPs. (B) SNPs with a specific characteristic (MAF, pop-MAF, distance to CODIS STR, and *D*’_avg_ conditions). (C) SNPs with two or more characteristics (combinations among MAF or pop-MAF, distance to CODIS STR, and *D*’_avg_ conditions). Full numerical results for the SNP selection strategies appear in Tables S5 and S6A.

### SNPs that possess a specific characteristic

We next investigated SNPs satisfying specific conditions on MAF, pop-MAF, distance to the CODIS STR, and *D*’_avg_ with the CODIS STR. We evaluated three conditions for each variable, more stringent conditions resulting in fewer remaining SNPs (Table S4). For each variable and condition, we considered random SNP sets among SNPs satisfying the condition (e.g., MAF ≥ 1%). In particular, we randomly selected 25, 50, 75, and 100 SNPs per STR, totaling 450, 900, 1350, and 1800 SNPs across 18 STRs; to assess variability across replicates, we performed this sampling 10 times.

**Fig. 2:**
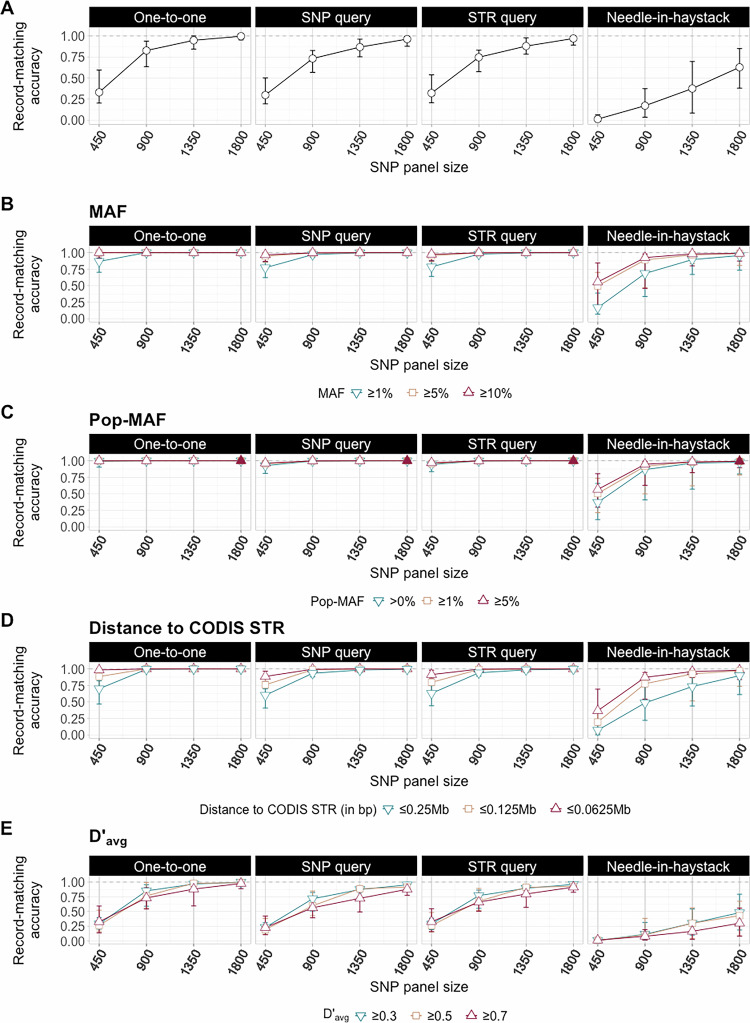
Record-matching accuracies for sets of SNPs that possess a specific characteristic. Rows correspond to different statistics used for SNP selection. **A** Random selection. **B** MAF. **C** Pop-MAF (minimal MAF across 1000 Genomes Project super-populations). **D** Distance between SNP and CODIS STR. **E** *D*’_avg_. Columns correspond to the four matching scenarios. Each plot shows record-matching accuracies for SNP sets of four sizes. Points represent the median, and error bars represent the range of record-matching accuracies across 100 replicates (10 partitions of the individuals, 10 SNP sets). Colors and point shapes correspond to different conditions. Closed symbols correspond to conditions that achieve comparable record-matching accuracies to those obtained with the full set of 192,672 SNPs. The full numerical values are shown in Table S5.

Figure 2 evaluates median record-matching accuracies for all conditions and all statistics (Fig. 2B–E), compared to random SNP selection (Fig. 2A). Each of the conditions based on MAF, pop-MAF, and distance enhances record-matching accuracies compared to random SNPs (Table S5). Record-matching accuracy increases with the stringency of the condition, especially for smaller SNP sets. Of the 9 MAF, pop-MAF, and distance conditions (3 statistics, 3 conditions), two produce comparable record-matching accuracies to the full set of SNPs with 1800 SNPs: pop-MAF ≥ 1% and pop-MAF ≥ 5%. Selecting SNPs with pop-MAF ≥ 1% produces median record-matching accuracies of 1 for one-to-one (range [1–1]), 1 for SNP query (range [0.997–1]), 1 for STR query (range [0.998–1]), and 0.992 for needle-in-haystack matching (range [0.784–1]). Selecting SNPs with pop-MAF ≥ 5% produces the same medians and ranges for one-to-one, SNP query, and STR query, and a different range for needle-in-haystack (median 0.992, range [0.896–1]) (Table S5C). These results suggest that 1800 carefully chosen SNPs suffice to match the record-matching accuracy of the full SNP set (Table 1B).

### SNPs that possess two or more specific characteristics

Finally, we examined SNPs that possess two or more characteristics. We considered the MAF, pop-MAF, and distance conditions that achieved comparable record-matching accuracy to the full data with smaller SNP sets than were seen with random SNPs in previous analyses.

**Fig. 3:**
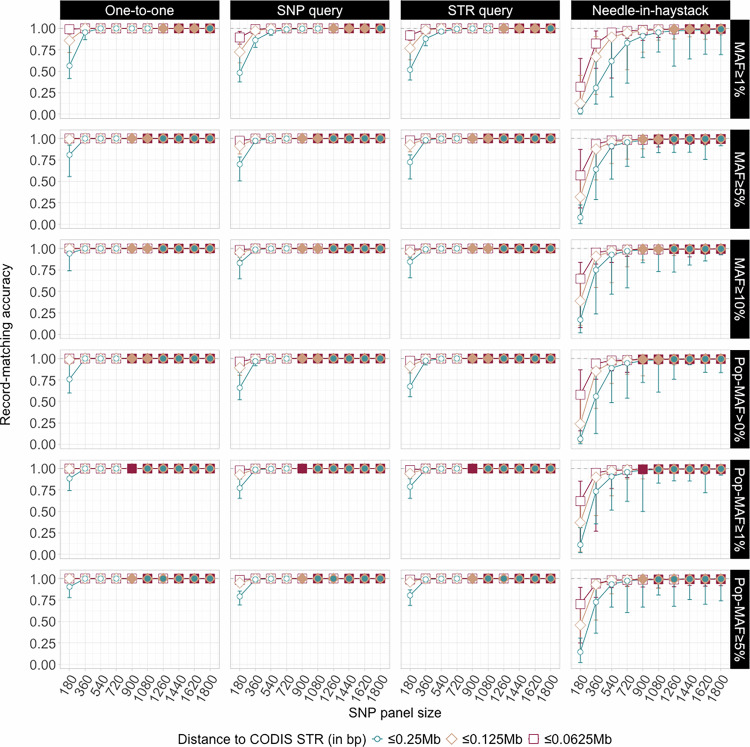
Record-matching accuracies for sets of SNPs that possess two characteristics, a MAF or pop-MAF condition and a condition on distance to the CODIS STR. Each row corresponds to a MAF or pop-MAF condition. Colors and point shapes represent different distance conditions. Columns represent the four matching scenarios. Each plot shows record-matching accuracies for SNP sets of 10 sizes, across 100 replicates (10 partitions of individuals, 10 SNP sets); points represent the median, and error bars represent the range. Closed symbols correspond to conditions that achieve comparable record-matching accuracies to those obtained with the full set of 192,672 SNPs. The full numerical values are shown in Table S6A.

Figure 3 examines the median record-matching accuracy of 18 combinations of MAF or pop-MAF and distance conditions (6×3). Six produce minimal sets of size 900 SNPs: (MAF ≥ 5%, distance ≤ 0.125 Mb), (MAF ≥ 10%, distance ≤ 0.125 Mb), (pop-MAF > 0%, distance ≤ 0.0625 Mb), (pop-MAF > 0%, distance ≤ 0.125 Mb), (pop-MAF ≥ 1%, distance ≤ 0.0625 Mb), and (pop-MAF ≥ 5%, distance ≤ 0.125 Mb) (Table S6A). Among the other 12 combinations, seven require 1080 SNPs, three require 1260, one requires 1440, and one requires 1800.

In Fig. 2E, selecting SNPs based on a *D*’_avg_ condition did not improve record-matching accuracy over random SNPs. Here, we explore the three *D*’_avg_ conditions in combination with the six allele-frequency conditions, three distance conditions, or both, considering only the 16 of 81 combinations that resulted in at least 100 SNPs per CODIS STR (Fig. S2, Table S6B). Across combinations, one (MAF ≥ 1%, distance ≤ 0.125 Mb, *D*’_avg_ ≥ 0.3) resulted in comparable record-matching accuracy to the full data with 1440 SNPs, another with 1620 SNPs, and two with 1800 SNPs. None did so with 900 SNPs.

In summary, combinations with three conditions, which reduce the number of available SNPs compared to use of two conditions, did not perform as well as the highest-performing combinations with two conditions. Selecting SNPs based on the highest-performing pairs, a MAF or pop-MAF condition and a maximal distance to the CODIS STR, can reduce the SNP set required for comparable record-matching accuracy to that achieved by the full set of 192,672 SNPs to 900 SNPs, or ~0.5% of the SNPs available in our dataset (Table 1C).

## Discussion

This article advances toward a minimal set of SNPs for accurate record-matching between SNP profiles and STR profiles. Although at least 9000 randomly selected SNPs were required for achieving record-matching accuracy comparable to that of the full set of 192,672 SNPs (Fig. 1), SNP sets chosen based on an allele frequency threshold in each of five super-populations (pop-MAF ≥ 1% or pop-MAF ≥ 5%) reduced the required number to 1800 (Fig. 2C, Table 1B). SNPs based on a MAF or pop-MAF condition and a distance condition produced a further reduction to 900 (Fig. 3, Table 1C): fewer than 1000 carefully selected SNPs suffice to enable record-matching at the level of the full data, and multiple SNP sets achieve this outcome.

Among the four scenarios we considered, the needle-in-haystack scenario is of greatest interest for practical queries. With the full set of 192,672 SNPs, the median record-matching accuracy for the needle-in-haystack scenario was 0.998 (range [0.987–1]) (Table S3); for 9000 randomly selected SNPs, the corresponding accuracy was 0.995 (range [0.962–1]) (Table 1A). With pop-MAF ≥ 1% and pop-MAF ≥ 5% selection criteria, sets of 1800 SNPs produced median accuracy 0.992 (with ranges [0.784–1] and [0.896–1], respectively; Table 1B). The six combinations of conditions that were most promising in sets of 900 SNPs—(MAF ≥ 5%, distance ≤ 0.125 Mb), (MAF ≥ 10%, distance ≤ 0.125 Mb), (pop-MAF > 0%, distance ≤ 0.0625 Mb), (pop-MAF > 0%, distance ≤ 0.125 Mb), (pop-MAF ≥ 1%, distance ≤ 0.0625 Mb), and (pop-MAF ≥ 5%, distance ≤ 0.125 Mb)—produced median accuracies of 0.990, 0.992, 0.990, 0.990, 0.992, and 0.994, and ranges of [0.879–1], [0.866–1], [0.939–1], [0.799–1], [0.936–1], and [0.949–1], respectively (Table 1C). Among these six, (pop-MAF ≥ 5%, distance ≤ 0.125 Mb) has both the highest median accuracy and the narrowest range (median 0.994, range [0.949–1]).

For a SNP with extremely low MAF, the training set may contain too few copies of the minor allele for BEAGLE to accurately learn the correlations between the SNP alleles and the alleles of its highly polymorphic neighboring STR. Hence, it is sensible that enforcing a MAF or pop-MAF minimum—so that the minor allele is likely to be sufficiently common in the training set—contributes to reducing the required size of SNP sets.

For the distance to the CODIS STR, the optimal distance within which to select SNPs remains uncertain. For a maximal distance set to a high number, many SNPs chosen within an associated window likely have little correlation with the CODIS STR, and provide little information for record-matching. For a maximal distance set to a low number, however, the number of available SNPs is smaller, and possibly insufficient for accurate record-matching. The panels that achieved the most accurate record-matching with sets of 900 SNPs included panels with a maximal distance of 0.0625 Mb and panels with a maximal distance of 0.125 Mb.

Sets of SNPs selected for high *D*’_avg_—high LD with the CODIS STR—produced comparatively low record-matching accuracies (Fig. 2E, Table S5). Furthermore, more stringent *D*’_avg_ thresholds led to lower accuracies. A possible explanation for these observations is that SNPs in high LD with the CODIS STR may often be in high LD with each other, and therefore somewhat redundant in their information for record-matching. Further analysis of LD-based criteria could help to illuminate this structure.

We note that our evaluation focused on 18 CODIS STRs, and a suitable SNP set for use in record-matching with the full 20 CODIS STRs would likely be larger by a factor of approximately 20/18. The SNP set could likely be reduced, however, by algorithmic procedures that select SNPs from a combinatorial space of possibilities, as was done in the “SNP tagging” problem [18–20], rather than random selection of SNPs among those that achieve specified thresholds.

We have focused on evaluating record-matching accuracy in the full set of individuals, rather than considering populations separately. In Fig. S3, for the six best-performing combinations of conditions, we separate record-matching accuracy by super-population. It was previously noted that varying genetic diversities across populations contribute to numerical values of match scores both for true matches and for non-matches, so that record-matching accuracy computed from match-score matrices does not have a simple dependence on such quantities [9]. In accord with [9], we find that record-matching accuracy is similar across super-populations; the order from greatest to smallest accuracy is AFR, AMR, EAS, EUR, SAS (Table S7). The pop-MAF conditions in many analyses ensure that selected SNPs are variable in and therefore useful for record-matching in multiple super-populations.

We can extrapolate from the 2504 individuals here to larger populations. Following an approach of [9] and [11], continuing with the six combinations of conditions, we evaluated the fraction of true matches whose match scores exceeded the minimal match score for achieving desired values for the “ratio of posterior and prior odds” statistic — thereby estimating the fraction of true matches that are possible to identify at a specified stringency level (e.g. a large population requiring a high ratio). The posterior odds is the likelihood ratio for the probability that individual A with STR profile R_A_ and individual B with SNP profile B with STR profile S_B_ are the same individual and the probability that they are unrelated. The prior odds is the probability that two individuals are the same individual in the absence of data; [9] and [11] consider this quantity as the reciprocal of the population size. The fractions of values exceeding thresholds for the ratio of posterior and prior odds (Table S8) were generally similar to or larger than corresponding fractions in the analysis in Table 2 of [9], with ~70% of true positive matches detectable at a ratio of 10^10^ with 900 SNPs (~90% of true positive matches at ratio 10^7^, 40% at 10^13^). In other words, the probability is relatively high that for carefully chosen sets of 900 SNPs, a true match could be identified by record-matching with high confidence in a large population. That this probability often exceeds the value from [9] is likely attributable to our considering 18 rather than 15 STRs (Table S8).

The study contributes to the possibility of producing systems for SNP-based forensic profiling that could eventually replace STR systems. Desirable SNP sets for such potential systems would include SNPs chosen in multiple forensically relevant classes, particularly SNPs variable in many populations and broadly useful in individual identification [5], and SNPs from existing incipient identification panels [8]. In addition to such SNPs, inclusion of fewer than 1000 SNPs could be used to support backward compatibility with the CODIS STRs via record-matching. An eventual panel could combine SNPs optimized for separate criteria or could include one set of SNPs optimized for multiple criteria simultaneously.

In choosing potential SNP panels for practical use, a strategy consistent with the design of the CODIS STRs is to prioritize SNPs that seek phenotypic privacy in their direct phenotypic associations, with minimal ability to predict phenotypes from allelic types. Such SNPs would attempt to support individual identification without revealing biomedical risks or other individual traits associated with a SNP profile. Record-matching inherently involves privacy tradeoffs, as it enables identifications between databases, including unexpected or undesirable linkages [9–11]. With combinatorial optimization approaches to further reduce the number of SNPs required for record-matching, some risks of a record-matching SNP panel can be mitigated; for example, the risk of unwanted phenotypic associations is lower in a smaller SNP set.

This study shows that the record-matching technique, which connected SNP and STR profiles with high accuracy in our previous studies with hundreds or tens of thousands of SNPs, can be conducted with comparable accuracy in SNP panels with fewer than 1000 SNPs. With additional optimization steps, the required number of SNPs could likely be further reduced. We caution, however, that we have focused on overall accuracy; we have not examined record-matching accuracy for specific individuals across different SNP panels, nor have we studied effects of SNP panel choice on specific match score values. In further evaluations of potential record-matching SNP panels, it will be important to assess many different performance measures, and to do so in larger sets of individuals than the 2504 considered here.

## Supplementary information

Supplemental Material

## Data Availability

Data in the form of phased haplotypes are available at https://github.com/tamigj/codis_panel/data/raw/. Code for performing the analyses is available at https://github.com/tamigj/codis_panel, including code to download and process data from [12] to produce the phased haplotypes used in the study.

## References

[1] Budowle B, van Daal A. Forensically relevant SNP classes. BioTechniques. 2008;44:603–10. 18474034 10.2144/000112806

[2] Novroski NM, Cihlar JC. Evolution of single-nucleotide polymorphism use in forensic genetics. Wiley Interdisc Rev Forensic Sci. 2022;4:e1459.

[3] Kayser M, Sajantila A, Butler JM, Parson W, Salas A, Gill P, et al. Forensic genetics: unde venisti et quo vadis?. Forensic Sci Int Genet. 2023;65:102881. 37173159 10.1016/j.fsigen.2023.102881

[4] Phillips C, Salas A, Sánchez JJ, Fondevila M, Gómez-Tato A, Álvarez-Dios J, et al. Inferring ancestral origin using a single multiplex assay of ancestry-informative marker SNPs. Forensic Sci Int Genet. 2007;1:273–80. 19083773 10.1016/j.fsigen.2007.06.008

[5] Pakstis AJ, Speed WC, Fang R, Hyland FC, Furtado MR, Kidd JR, et al. SNPs for a universal individual identification panel. Hum Genet. 2010;127:315–24. 19937056 10.1007/s00439-009-0771-1

[6] Gettings KB, Lai R, Johnson JL, Peck MA, Hart JA, Gordish-Dressman H, et al. A 50-SNP assay for biogeographic ancestry and phenotype prediction in the U.S. population. Forensic Sci Int Genet. 2014;8:101–8. 24315596 10.1016/j.fsigen.2013.07.010

[7] Larmuseau MHD, Van Geystelen A, Kayser M, van Oven M, Decorte R. Towards a consensus Y-chromosomal phylogeny and Y-SNP set in forensics in the next-generation sequencing era. Forensic Sci Int Genet. 2015;15:39–42. 25488610 10.1016/j.fsigen.2014.11.012

[8] Tillmar A, Sturk-Andreaggi K, Daniels-Higginbotham J, Thomas JT, Marshall C. The FORCE panel: an all-in-one SNP marker set for confirming investigative genetic genealogy leads and for general forensic applications. Genes. 2021;12:1968. 34946917 10.3390/genes12121968 PMC8702142

[9] Edge MD, Algee-Hewitt BFB, Pemberton TJ, Li JZ, Rosenberg NA. Linkage disequilibrium matches forensic genetic records to disjoint genomic marker sets. Proc Natl Acad Sci USA. 2017;114:5671–6. 28507140 10.1073/pnas.1619944114 PMC5465933

[10] Kim J, Edge MD, Algee-Hewitt BFB, Li JZ, Rosenberg NA. Statistical detection of relatives typed with disjoint forensic and biomedical loci. Cell. 2018;175:848–58. 30318150 10.1016/j.cell.2018.09.008 PMC6240431

[11] Kim J, Rosenberg NA. Record-matching of STR profiles with fragmentary genomic SNP data. Eur J Hum Genet. 2023;31:1283–90. 37567955 10.1038/s41431-023-01430-9 PMC10620386

[12] Saini S, Mitra I, Mousavi N, Fotsing SF, Gymrek M. A reference haplotype panel for genome-wide imputation of short tandem repeats. Nat Comm. 2018;9:4397. 10.1038/s41467-018-06694-0 PMC6199332 30353011

[13] Hares DR. Selection and implementation of expanded CODIS core loci in the United States. Forensic Sci Int Genet. 2015;17:33–34. 25797140 10.1016/j.fsigen.2015.03.006

[14] 1000 Genomes Project Consortium. A global reference for human genetic variation. Nature. 2015;526:68–74. 26432245 10.1038/nature15393 PMC4750478

[15] Browning SR, Browning BL. Rapid and accurate haplotype phasing and missing-data inference for whole-genome association studies by use of localized haplotype clustering. Am J Hum Genet. 2007;81:1084–97. 17924348 10.1086/521987 PMC2265661

[16] Kuhn HW. The Hungarian method for the assignment problem. Nav Res Logist Q. 1955;2:83–97.

[17] Payseur BA, Place M, Weber JL. Linkage disequilibrium between STRPs and SNPs across the human genome. Am J Hum Genet. 2008;82:1039–50. 18423524 10.1016/j.ajhg.2008.02.018 PMC2427224

[18] Carlson CS, Eberle MA, Rieder MJ, Yi Q, Kruglyak L, Nickerson DA. Selecting a maximally informative set of single-nucleotide polymorphisms for association analyses using linkage disequilibrium. Am J Hum Genet. 2004;74:106–20. 14681826 10.1086/381000 PMC1181897

[19] Stram DO. Tag SNP selection for association studies. Genet Epidemiol. 2004;27:365–74. 15372618 10.1002/gepi.20028

[20] Ke X, Miretti MM, Broxholme J, Hunt S, Beck S, Bentley DR, et al. A comparison of tagging methods and their tagging space. Hum Mol Genet. 2005;14:2757–67. 16103130 10.1093/hmg/ddi309

