# Supplementary material for: Toward minimal SNP sets for record-matching with CODIS STR profiles
Source: Eur J Hum Genet. 2025 Sep 22;34(8):1097–103. doi: 10.1038/s41431-025-01941-7 (PMC13424081; doi:10.1038/s41431-025-01941-7)
Supplement: Supplementary file 1 — Supplemental Material [file 41431_2025_1941_MOESM1_ESM.pdf]

Supplemental Material

Toward minimal SNP sets for record-matching with CODIS STR profiles

Tamara Gjorgjieva, Noah A. Rosenberg

Supplementary Figures

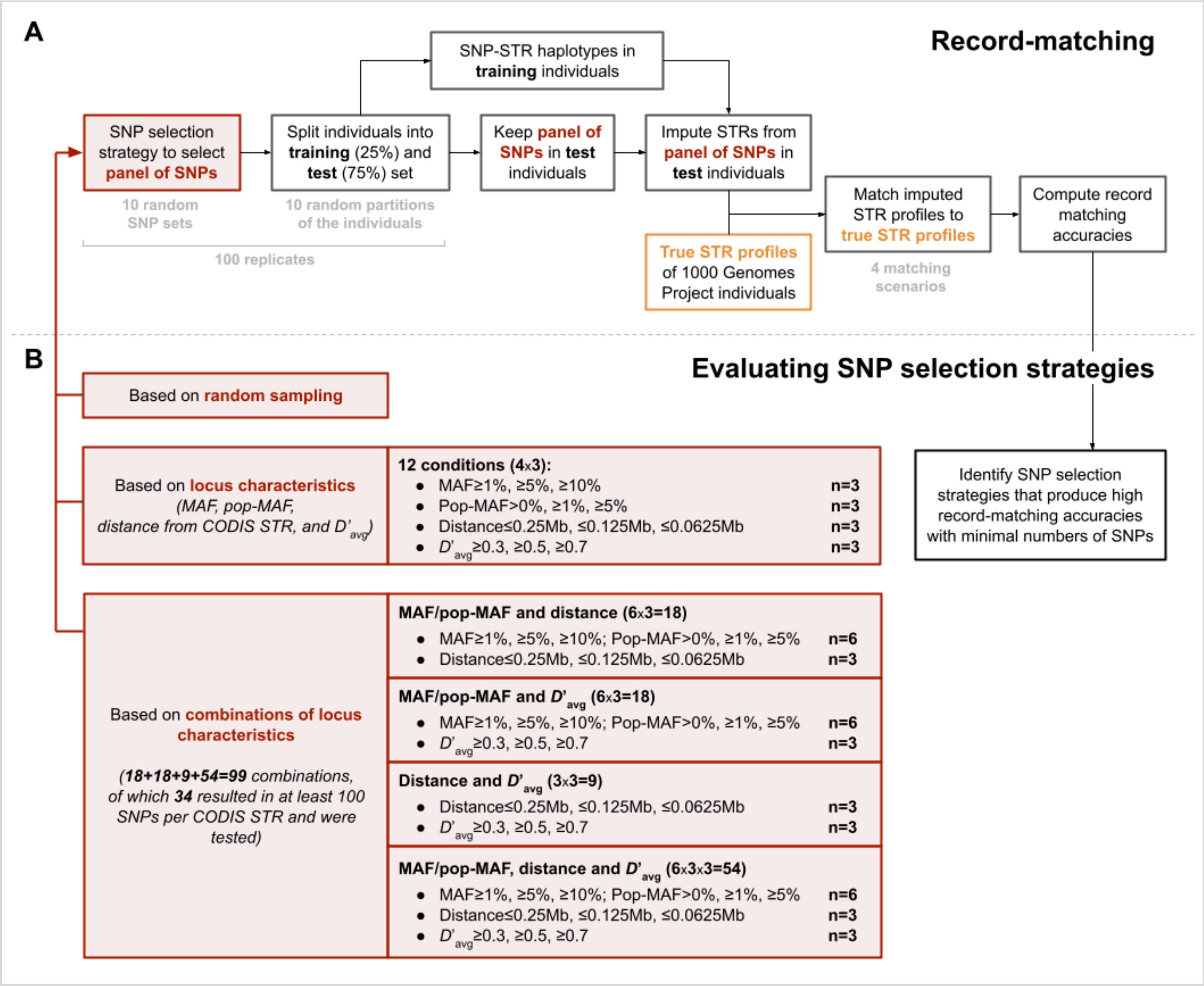

Figure S1. SNP selection and record-matching pipeline. (A) Pipeline for evaluating record-matching accuracy for a set of SNPs in the 1000 Genomes Project data. (B) SNP selection strategies.

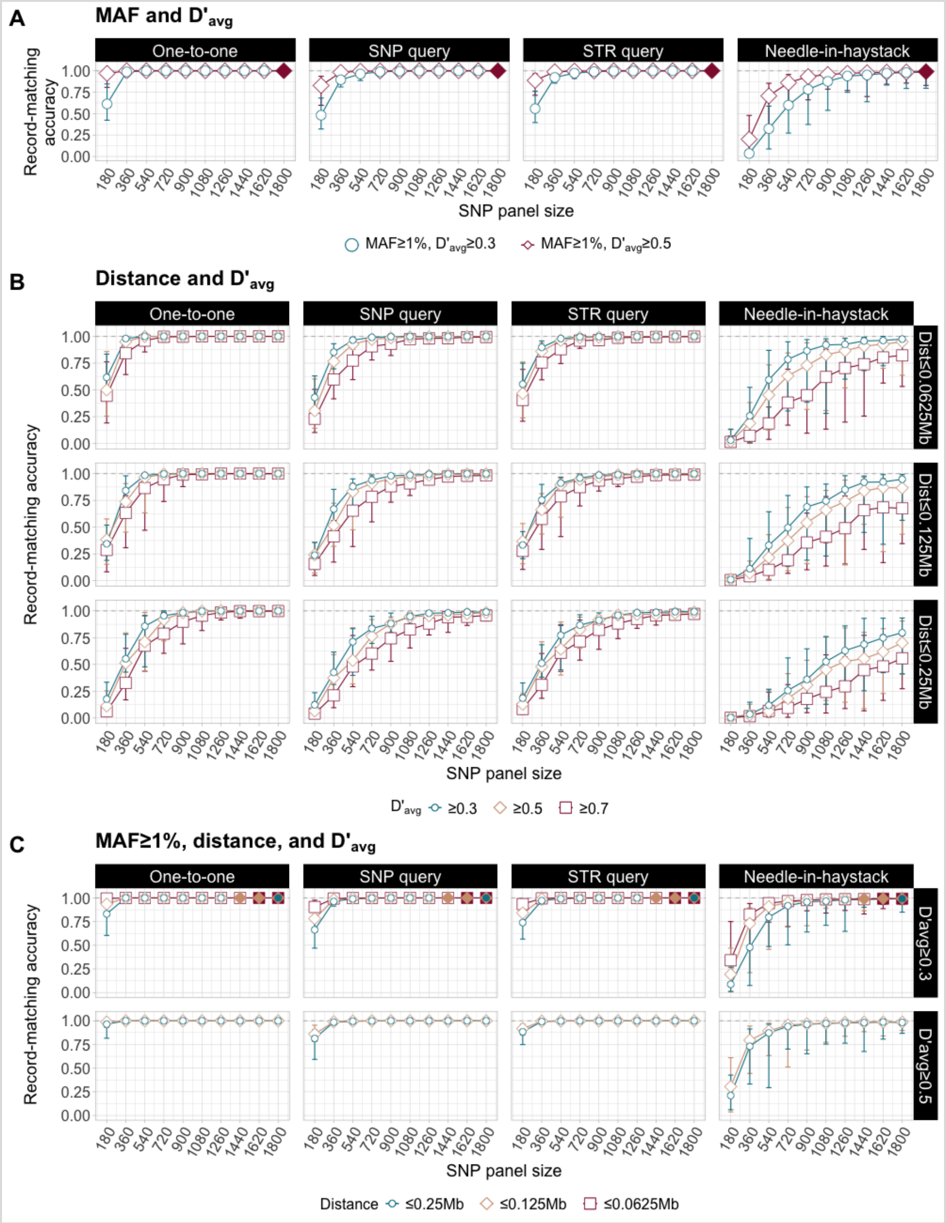

Figure S2. Record-matching accuracies for sets of SNPs selected based on combinations of MAF or pop-MAF, distance, and D'_{avg} filtering conditions. (A) Combinations of MAF or pop-MAF and D'_{avg}, with colors corresponding to different combinations. (B) Combinations of distance and D'_{avg}. Rows correspond to different distance conditions, and colors and shapes to different D'_{avg} conditions. (C) Combinations of MAF or pop-MAF, distance, and D'_{avg}, with colors and shapes corresponding to different combinations. Each plot shows matching accuracies for 10 SNP panel sizes. Closed symbols correspond to SNP sets that achieve comparable record-matching accuracies to those obtained with the full set of 192,672 SNPs. Points represent the median, and error bars show the range across 100 replicates. Full numerical results are shown in Table S6B.

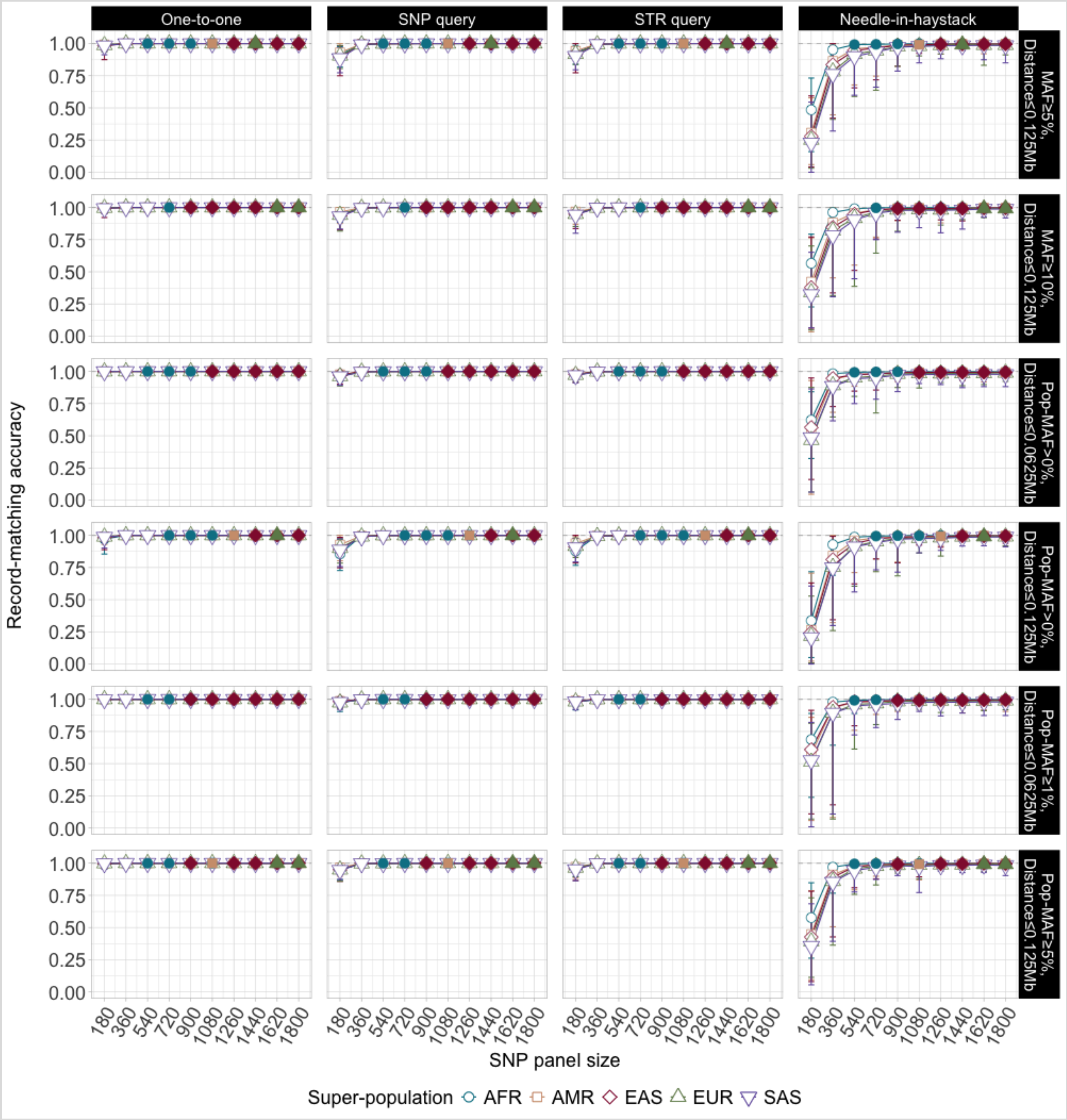

Figure S3. Record-matching accuracies in five super-populations for the six conditions in Table 1C that achieved record-matching accuracy in the full set of individuals comparable to that achieved with the full SNP set. Rows correspond to the six conditions, and colors and shapes to super-populations. Each plot shows matching accuracies for 10 SNP panel sizes. Closed symbols correspond to SNP sets that, for a given super-population, achieve median record-matching accuracies in the one-to-one, one-to-many with SNP query, one-to-many with STR query, and needle-in-haystack scenarios of 1, 1, 1, and ≥0.99. Points represent the median, and error bars show the range across 100 replicates. Full numerical results are shown in Table S7.

Supplementary Tables

STR	Number of SNPs	Used in Kim & Rosenberg (2023)?
CSF1PO	9905	Yes
D10S1248	12,021	Yes
D12S391	10,243	No
D13S317	9253	Yes
D18S51	9510	Yes
D19S433	10,617	Yes
D1S1656	10,618	No
D22S1045	11,282	Yes
D2S1338	9843	No
D2S441	10,363	Yes
D3S1358	9427	Yes
D5S818	9170	Yes
D7S820	9569	Yes
D8S1179	9994	Yes
FGA	9360	Yes
TH01	11,990	Yes
TPOX	18,330	Yes
vWA	11,177	Yes
Total	192,672	15

Table S1. Number of SNP loci in 1-Mb windows around 18 CODIS STRs.

	MAF	Pop-MAF	Distance	D'_{avg}	Total number of SNPs across STRs	Minimal number of SNPs across STRs	Tested?
**A. MAF and distance combinations (*n*=18)**							
	1%		0.0625 Mb		10,421	373	Yes
	1%		0.125 Mb		20,122	818	Yes
	1%		0.25 Mb		40,738	1615	Yes
	5%		0.0625 Mb		6300	224	Yes
	5%		0.125 Mb		11,829	439	Yes
	5%		0.25 Mb		23,844	979	Yes
	10%		0.0625 Mb		4945	173	Yes
	10%		0.125 Mb		9152	332	Yes
	10%		0.25 Mb		18,629	721	Yes
		0%	0.0625 Mb		6600	211	Yes
		0%	0.125 Mb		12,196	466	Yes
		0%	0.25 Mb		25,024	889	Yes
		1%	0.0625 Mb		5247	161	Yes
		1%	0.125 Mb		9579	263	Yes
		1%	0.25 Mb		19,401	572	Yes
		5%	0.0625 Mb		3675	109	Yes
		5%	0.125 Mb		6726	241	Yes
		5%	0.25 Mb		13,790	474	Yes
**B. MAF, distance, and D'_{avg} combinations (*n*=81)**							
	1%		0.0625 Mb	0.3	7657	168	Yes
	1%		0.0625 Mb	0.5	4805	77	No
	1%		0.0625 Mb	0.7	2287	12	No
	1%		0.125 Mb	0.3	12,637	277	Yes
	1%		0.125 Mb	0.5	6950	113	Yes
	1%		0.125 Mb	0.7	3085	17	No
	1%		0.25 Mb	0.3	19,668	358	Yes
	1%		0.25 Mb	0.5	8914	126	Yes
	1%		0.25 Mb	0.7	3549	17	No
	5%		0.0625 Mb	0.3	3987	24	No
	5%		0.0625 Mb	0.5	2051	2	No
	5%		0.0625 Mb	0.7	876	0	No

	5%		0.125 Mb	0.3	5738	32	No
	5%		0.125 Mb	0.5	2732	2	No
	5%		0.125 Mb	0.7	1228	0	No
	5%		0.25 Mb	0.3	7419	32	No
	5%		0.25 Mb	0.5	3098	2	No
	5%		0.25 Mb	0.7	1374	0	No
	10%		0.0625 Mb	0.3	3000	8	No
	10%		0.0625 Mb	0.5	1521	0	No
	10%		0.0625 Mb	0.7	730	0	No
	10%		0.125 Mb	0.3	4035	8	No
	10%		0.125 Mb	0.5	1887	0	No
	10%		0.125 Mb	0.7	896	0	No
	10%		0.25 Mb	0.3	4966	8	No
	10%		0.25 Mb	0.5	2105	0	No
	10%		0.25 Mb	0.7	976	0	No
		0%	0.0625 Mb	0.3	4217	34	No
		0%	0.0625 Mb	0.5	2243	9	No
		0%	0.0625 Mb	0.7	968	0	No
		0%	0.125 Mb	0.3	6057	44	No
		0%	0.125 Mb	0.5	2989	10	No
		0%	0.125 Mb	0.7	1307	0	No
		0%	0.25 Mb	0.3	7911	51	No
		0%	0.25 Mb	0.5	3436	10	No
		0%	0.25 Mb	0.7	1469	0	No
		1%	0.0625 Mb	0.3	3209	12	No
		1%	0.0625 Mb	0.5	1672	2	No
		1%	0.0625 Mb	0.7	760	0	No
		1%	0.125 Mb	0.3	4322	12	No
		1%	0.125 Mb	0.5	2051	2	No
		1%	0.125 Mb	0.7	922	0	No
		1%	0.25 Mb	0.3	5263	12	No
		1%	0.25 Mb	0.5	2272	2	No
		1%	0.25 Mb	0.7	994	0	No
		5%	0.0625 Mb	0.3	2215	4	No
		5%	0.0625 Mb	0.5	1124	0	No
		5%	0.0625 Mb	0.7	502	0	No
		5%	0.125 Mb	0.3	2858	4	No

		5%	0.125 Mb	0.5	1373	0	No
		5%	0.125 Mb	0.7	629	0	No
		5%	0.25 Mb	0.3	3338	4	No
		5%	0.25 Mb	0.5	1538	0	No
		5%	0.25 Mb	0.7	686	0	No
	1%			0.3	28,940	403	Yes
	1%			0.5	10,099	126	Yes
	1%			0.7	3635	17	No
	5%			0.3	8752	32	No
	5%			0.5	3147	2	No
	5%			0.7	1375	0	No
	10%			0.3	5481	8	No
	10%			0.5	2126	0	No
	10%			0.7	976	0	No
		0%		0.3	9211	55	No
		0%		0.5	3551	11	No
		0%		0.7	1482	0	No
		1%		0.3	5467	12	No
		1%		0.5	2278	2	No
		1%		0.7	994	0	No
		5%		0.3	3455	4	No
		5%		0.5	1544	0	No
		5%		0.7	686	0	No
			0.0625 Mb	0.3	21,237	847	Yes
			0.0625 Mb	0.5	17,348	667	Yes
			0.0625 Mb	0.7	12,766	431	Yes
			0.125 Mb	0.3	39,038	1619	Yes
			0.125 Mb	0.5	30,398	1174	Yes
			0.125 Mb	0.7	21,493	727	Yes
			0.25 Mb	0.3	71,141	2886	Yes
			0.25 Mb	0.5	51,811	1983	Yes
			0.25 Mb	0.7	34,874	1177	Yes

Table S2. Combinations of locus characteristics. (A) Combinations that include one of six MAF or pop-MAF conditions and one of three distance conditions (n=6×3=18). (B) MAF or pop-MAF, distance, and D'_{avg} conditions (n=81; 6×3×3=54 combinations including a MAF or pop-MAF condition, a distance condition, and a D'_{avg} condition, 6×3=18 combinations including a MAF or pop-MAF condition and a D'_{avg} condition, and 3×3=9 combinations including a distance condition and a D'_{avg} condition).

	One-to-one		SNP query		STR query		Needle-in-haystack	
SNP panel size	Median	Range	Median	Range	Median	Range	Median	Range
180	0.050	[0.014 - 0.086]	0.043	[0.011 - 0.081]	0.054	[0.024 - 0.099]	0	[0 - 0.006]
450	0.331	[0.203 - 0.594]	0.298	[0.193 - 0.502]	0.321	[0.208 - 0.538]	0.013	[0 - 0.064]
900	0.827	[0.636 - 0.938]	0.732	[0.567 - 0.827]	0.747	[0.575 - 0.831]	0.172	[0.035 - 0.374]
1350	0.948	[0.843 - 1]	0.867	[0.752 - 0.962]	0.879	[0.783 - 0.978]	0.375	[0.085 - 0.696]
1800	0.997	[0.952 - 1]	0.962	[0.877 - 0.992]	0.970	[0.891 - 0.995]	0.628	[0.380 - 0.850]
2250	1	[0.994 - 1]	0.981	[0.958 - 0.997]	0.984	[0.965 - 1]	0.764	[0.358 - 0.930]
4500	1	[1 - 1]	0.998	[0.992 - 1]	1	[0.994 - 1]	0.962	[0.776 - 0.992]
9000	1	[1 - 1]	1	[0.997 - 1]	1	[0.998 - 1]	0.995	[0.962 - 1]
18,000	1	[1 - 1]	1	[0.998 - 1]	1	[0.998 - 1]	0.997	[0.982 - 1]
90,000	1	[1 - 1]	1	[0.998 - 1]	1	[0.998 - 1]	0.998	[0.989 - 1]
192,672	1	[1 - 1]	1	[0.998 - 1]	1	[1 - 1]	0.998	[0.987 - 1]

Table S3. Record-matching accuracies for sets of randomly selected SNPs. Rows highlighted in light pink correspond to SNP sets with matching accuracies comparable to those produced by the full set of 192,672 SNPs: a median of 1 for one-to-one, SNP query, and STR query, and a median≥0.99 for needle-in-haystack. The table shows medians and ranges plotted in Figure 1.

Statistic and condition	Number of SNPs after filtering
MAF≥1%	79,857
MAF≥5%	46,569
MAF≥10%	36,349
Pop-MAF>0%	49,265
Pop-MAF≥1%	37,683
Pop-MAF≥5%	27,007
Distance≤0.25Mb	97,294
Distance≤0.125Mb	48,641
Distance≤0.0625Mb	24,947
$D'_{avg}\geq0.3$	129,151
$D'_{avg}\geq0.5$	87,728
$D'_{avg}\geq0.7$	57,276

Table S4. Number of SNPs remaining after filtering based on various statistics and conditions.

		One-to-one		SNP query		STR query		Needle-in-haystack	
**Condition**	**SNP panel size**	**Median**	**Range**	**Median**	**Range**	**Median**	**Range**	**Median**	**Range**
**A. Random SNP selection**									
Random	450	0.331	[0.203 - 0.594]	0.298	[0.193 - 0.502]	0.321	[0.208 - 0.538]	0.013	[0 - 0.064]
	900	0.827	[0.636 - 0.938]	0.732	[0.567 - 0.827]	0.747	[0.575 - 0.831]	0.172	[0.035 - 0.374]
	1350	0.948	[0.843 - 1]	0.867	[0.752 - 0.962]	0.879	[0.783 - 0.978]	0.375	[0.085 - 0.696]
	1800	0.997	[0.952 - 1]	0.962	[0.877 - 0.992]	0.970	[0.891 - 0.995]	0.628	[0.380 - 0.850]
**B. MAF**									
MAF≥1%	450	0.867	[0.703 - 0.965]	0.772	[0.621 - 0.858]	0.784	[0.641 - 0.877]	0.166	[0.069 - 0.388]
	900	1	[0.974 - 1]	0.970	[0.927 - 0.989]	0.976	[0.936 - 0.994]	0.685	[0.335 - 0.885]
	1350	1	[0.997 - 1]	0.997	[0.974 - 1]	0.997	[0.984 - 1]	0.895	[0.669 - 0.971]
	1800	1	[0.997 - 1]	0.998	[0.992 - 1]	1	[0.994 - 1]	0.955	[0.733 - 0.990]
MAF≥5%	450	0.997	[0.947 - 1]	0.946	[0.851 - 0.981]	0.953	[0.866 - 0.992]	0.495	[0.181 - 0.701]
	900	1	[0.997 - 1]	0.995	[0.986 - 1]	0.997	[0.990 - 1]	0.889	[0.468 - 0.962]
	1350	1	[1 - 1]	0.998	[0.995 - 1]	1	[0.995 - 1]	0.965	[0.832 - 0.997]
	1800	1	[1 - 1]	1	[0.997 - 1]	1	[0.998 - 1]	0.987	[0.805 - 1]
MAF≥10%	450	1	[0.919 - 1]	0.968	[0.867 - 0.992]	0.974	[0.875 - 0.994]	0.555	[0.203 - 0.843]
	900	1	[0.997 - 1]	0.997	[0.987 - 1]	0.998	[0.990 - 1]	0.925	[0.458 - 0.981]
	1350	1	[1 - 1]	1	[0.995 - 1]	1	[0.997 - 1]	0.982	[0.800 - 0.998]
	1800	1	[1 - 1]	1	[0.997 - 1]	1	[0.998 - 1]	0.989	[0.872 - 1]
**C. Pop-MAF**									
Pop-MAF>0%	450	0.993	[0.903 - 1]	0.926	[0.808 - 0.966]	0.942	[0.835 - 0.973]	0.369	[0.109 - 0.657]
	900	1	[0.997 - 1]	0.995	[0.971 - 1]	0.997	[0.979 - 1]	0.866	[0.407 - 0.958]
	1350	1	[1 - 1]	0.998	[0.992 - 1]	1	[0.994 - 1]	0.963	[0.570 - 0.997]
	1800	1	[1 - 1]	1	[0.995 - 1]	1	[0.997 - 1]	0.978	[0.802 - 0.998]

	Pop-MAF≥1%	450	0.997	[0.982 - 1]	0.951	[0.899 - 0.976]	0.956	[0.923 - 0.982]	0.503	[0.214 - 0.733]
		900	1	[0.997 - 1]	0.997	[0.989 - 1]	0.998	[0.984 - 1]	0.917	[0.498 - 0.987]
		1350	1	[1 - 1]	1	[0.994 - 1]	1	[0.997 - 1]	0.979	[0.620 - 0.998]
		1800	1	[1 - 1]	1	[0.997 - 1]	1	[0.998 - 1]	0.992	[0.784 - 1]
	Pop-MAF≥5%	450	1	[0.987 - 1]	0.964	[0.931 - 0.995]	0.970	[0.944 - 1]	0.565	[0.297 - 0.804]
		900	1	[1 - 1]	0.998	[0.992 - 1]	0.999	[0.994 - 1]	0.947	[0.628 - 0.990]
		1350	1	[1 - 1]	1	[0.997 - 1]	1	[0.997 - 1]	0.982	[0.819 - 0.998]
		1800	1	[1 - 1]	1	[0.997 - 1]	1	[0.998 - 1]	0.992	[0.896 - 1]
**D. Distance to CODIS STR**										
	Distance≤0.0625Mb	450	0.984	[0.930 - 1]	0.886	[0.810 - 0.965]	0.914	[0.851 - 0.981]	0.366	[0.149 - 0.693]
		900	1	[0.997 - 1]	0.995	[0.982 - 1]	0.997	[0.981 - 1]	0.872	[0.543 - 0.946]
		1350	1	[1 - 1]	1	[0.995 - 1]	1	[0.995 - 1]	0.96	[0.867 - 0.997]
		1800	1	[1 - 1]	1	[0.995 - 1]	1	[0.997 - 1]	0.98	[0.839 - 0.997]
	Distance≤0.125Mb	450	0.884	[0.733 - 0.981]	0.759	[0.625 - 0.896]	0.796	[0.663 - 0.919]	0.194	[0.016 - 0.372]
		900	1	[0.997 - 1]	0.987	[0.971 - 0.998]	0.990	[0.974 - 1]	0.775	[0.454 - 0.915]
		1350	1	[1 - 1]	0.998	[0.990 - 1]	0.998	[0.992 - 1]	0.924	[0.514 - 0.992]
		1800	1	[1 - 1]	1	[0.994 - 1]	1	[0.995 - 1]	0.97	[0.736 - 0.998]
	Distance≤0.25Mb	450	0.699	[0.466 - 0.818]	0.601	[0.406 - 0.696]	0.629	[0.441 - 0.746]	0.071	[0.002 - 0.235]
		900	0.994	[0.957 - 1]	0.933	[0.879 - 0.966]	0.940	[0.890 - 0.978]	0.485	[0.220 - 0.730]
		1350	1	[0.989 - 1]	0.982	[0.944 - 0.998]	0.986	[0.946 - 0.995]	0.732	[0.438 - 0.895]
		1800	1	[1 - 1]	0.995	[0.981 - 1]	0.997	[0.984 - 1]	0.897	[0.610 - 0.979]
**E. D'_{avg} between SNP and CODIS STR**										
	D'_{avg}≥0.3	450	0.290	[0.200 - 0.398]	0.230	[0.150 - 0.310]	0.288	[0.192 - 0.387]	0.010	[0 - 0.030]
		900	0.851	[0.589 - 0.955]	0.716	[0.510 - 0.823]	0.770	[0.553 - 0.863]	0.114	[0.021 - 0.315]
		1350	0.966	[0.858 - 1]	0.874	[0.743 - 0.928]	0.895	[0.772 - 0.941]	0.301	[0.099 - 0.543]
		1800	0.997	[0.941 - 1]	0.952	[0.856 - 0.986]	0.960	[0.874 - 0.990]	0.480	[0.185 - 0.794]

	D'_{avg}≥0.5	450	0.262	[0.161 - 0.522]	0.192	[0.112 - 0.382]	0.260	[0.153 - 0.484]	0.011	[0 - 0.035]
		900	0.766	[0.553 - 0.994]	0.598	[0.428 - 0.848]	0.675	[0.518 - 0.891]	0.103	[0.018 - 0.383]
		1350	0.979	[0.874 - 1]	0.886	[0.744 - 0.941]	0.911	[0.789 - 0.963]	0.300	[0.040 - 0.564]
		1800	0.990	[0.954 - 1]	0.915	[0.861 - 0.970]	0.928	[0.887 - 0.982]	0.430	[0.088 - 0.674]
	D'_{avg}≥0.7	450	0.326	[0.137 - 0.593]	0.228	[0.105 - 0.423]	0.327	[0.158 - 0.548]	0.013	[0 - 0.050]
		900	0.731	[0.542 - 0.904]	0.563	[0.393 - 0.732]	0.660	[0.503 - 0.812]	0.079	[0.014 - 0.192]
		1350	0.881	[0.594 - 0.986]	0.724	[0.492 - 0.874]	0.798	[0.569 - 0.925]	0.164	[0.026 - 0.318]
		1800	0.976	[0.888 - 1]	0.877	[0.773 - 0.955]	0.914	[0.826 - 0.970]	0.301	[0.080 - 0.558]

Table S5. Record-matching accuracies for sets of SNPs that possess a specific characteristic. (A) Random selection. (B) MAF. (C) Pop-MAF. (D) Distance between SNP and CODIS STR. (E) D'_{avg}. Rows highlighted in light pink correspond to SNP sets with matching accuracies comparable to those produced by the full set of 192,672 SNPs: a median of 1 for one-to-one, SNP query and STR query, and a median≥0.99 for needle-in-haystack. The table shows medians and ranges plotted in Figure 2.

	Condition	SNP panel size	One-to-one Median	One-to-one Range	SNP query Median	SNP query Range	STR query Median	STR query Range	Needle-in-haystack Median	Needle-in-haystack Range
**A. MAF or pop-MAF and distance conditions (*n*=18)**										
	MAF≥1%, Distance≤0.0625Mb	180	0.99	[0.935 - 1]	0.894	[0.818 - 0.963]	0.919	[0.863 - 0.973]	0.319	[0.067 - 0.650]
		360	1	[0.997 - 1]	0.992	[0.973 - 1]	0.994	[0.979 - 1]	0.823	[0.535 - 0.970]
		540	1	[1 - 1]	0.998	[0.992 - 1]	1	[0.997 - 1]	0.952	[0.422 - 0.994]
		720	1	[1 - 1]	1	[0.995 - 1]	1	[0.997 - 1]	0.97	[0.887 - 0.994]
		900	1	[1 - 1]	1	[0.998 - 1]	1	[0.998 - 1]	0.983	[0.861 - 1]
		1080	1	[1 - 1]	1	[0.997 - 1]	1	[0.998 - 1]	0.989	[0.895 - 1]
		1260	1	[1 - 1]	1	[0.998 - 1]	1	[0.998 - 1]	0.989	[0.935 - 1]
		1440	1	[1 - 1]	1	[0.998 - 1]	1	[0.998 - 1]	0.992	[0.952 - 1]
		1620	1	[1 - 1]	1	[0.998 - 1]	1	[0.998 - 1]	0.994	[0.936 - 1]
		1800	1	[1 - 1]	1	[0.997 - 1]	1	[0.998 - 1]	0.994	[0.950 - 1]
	MAF≥1%, Distance≤0.125Mb	180	0.859	[0.719 - 0.997]	0.725	[0.599 - 0.883]	0.768	[0.634 - 0.922]	0.127	[0.021 - 0.452]
		360	1	[0.990 - 1]	0.973	[0.936 - 0.997]	0.979	[0.938 - 0.997]	0.670	[0.232 - 0.906]
		540	1	[1 - 1]	0.997	[0.986 - 1]	0.998	[0.989 - 1]	0.899	[0.649 - 0.976]
		720	1	[1 - 1]	0.998	[0.995 - 1]	1	[0.997 - 1]	0.949	[0.519 - 0.989]
		900	1	[1 - 1]	1	[0.997 - 1]	1	[0.997 - 1]	0.971	[0.722 - 0.995]
		1080	1	[1 - 1]	1	[0.997 - 1]	1	[0.997 - 1]	0.985	[0.863 - 1]
		1260	1	[1 - 1]	1	[0.998 - 1]	1	[0.998 - 1]	0.990	[0.877 - 1]
		1440	1	[1 - 1]	1	[0.997 - 1]	1	[0.998 - 1]	0.992	[0.901 - 1]
		1620	1	[1 - 1]	1	[0.998 - 1]	1	[0.998 - 1]	0.995	[0.872 - 1]
		1800	1	[1 - 1]	1	[0.998 - 1]	1	[0.998 - 1]	0.995	[0.925 - 1]
	MAF≥1%,	180	0.564	[0.415 - 0.843]	0.482	[0.377 - 0.720]	0.518	[0.398 - 0.746]	0.038	[0.002 - 0.153]

	Distance≤0.25Mb	360	0.954	[0.869 - 1]	0.863	[0.778 - 0.941]	0.879	[0.799 - 0.955]	0.308	[0.118 - 0.617]
		540	1	[0.979 - 1]	0.959	[0.919 - 0.995]	0.964	[0.938 - 0.997]	0.619	[0.201 - 0.859]
		720	1	[0.997 - 1]	0.989	[0.974 - 1]	0.994	[0.976 - 1]	0.831	[0.361 - 0.952]
		900	1	[1 - 1]	0.997	[0.987 - 1]	0.998	[0.989 - 1]	0.911	[0.660 - 0.976]
		1080	1	[1 - 1]	0.998	[0.992 - 1]	1	[0.995 - 1]	0.954	[0.724 - 0.989]
		1260	1	[1 - 1]	1	[0.994 - 1]	1	[0.995 - 1]	0.973	[0.561 - 0.997]
		1440	1	[1 - 1]	1	[0.997 - 1]	1	[0.997 - 1]	0.976	[0.644 - 0.998]
		1620	1	[1 - 1]	1	[0.997 - 1]	1	[0.998 - 1]	0.984	[0.698 - 0.998]
		1800	1	[1 - 1]	1	[0.998 - 1]	1	[0.998 - 1]	0.990	[0.695 - 1]
	MAF≥5%, Distance≤0.0625Mb	180	1	[0.989 - 1]	0.973	[0.939 - 0.989]	0.978	[0.939 - 0.998]	0.569	[0.190 - 0.872]
		360	1	[1 - 1]	0.998	[0.989 - 1]	1	[0.990 - 1]	0.935	[0.661 - 0.984]
		540	1	[1 - 1]	1	[0.997 - 1]	1	[0.997 - 1]	0.978	[0.879 - 0.997]
		720	1	[1 - 1]	1	[0.998 - 1]	1	[0.998 - 1]	0.984	[0.931 - 1]
		900	1	[1 - 1]	1	[0.997 - 1]	1	[0.998 - 1]	0.986	[0.930 - 1]
		1080	1	[1 - 1]	1	[0.998 - 1]	1	[0.998 - 1]	0.990	[0.901 - 1]
		1260	1	[1 - 1]	1	[0.998 - 1]	1	[0.998 - 1]	0.994	[0.928 - 1]
		1440	1	[1 - 1]	1	[0.998 - 1]	1	[0.998 - 1]	0.994	[0.931 - 1]
		1620	1	[1 - 1]	1	[0.998 - 1]	1	[0.998 - 1]	0.992	[0.933 - 1]
		1800	1	[1 - 1]	1	[0.998 - 1]	1	[0.998 - 1]	0.994	[0.939 - 1]
	MAF≥5%, Distance≤0.125Mb	180	0.991	[0.942 - 1]	0.912	[0.819 - 0.946]	0.927	[0.845 - 0.963]	0.318	[0.065 - 0.554]
		360	1	[0.997 - 1]	0.995	[0.986 - 1]	0.997	[0.987 - 1]	0.876	[0.516 - 0.984]
		540	1	[1 - 1]	1	[0.995 - 1]	1	[0.997 - 1]	0.957	[0.708 - 0.995]
		720	1	[1 - 1]	1	[0.997 - 1]	1	[0.997 - 1]	0.975	[0.759 - 0.997]
		900	1	[1 - 1]	1	[0.998 - 1]	1	[0.997 - 1]	0.990	[0.879 - 1]
		1080	1	[1 - 1]	1	[0.998 - 1]	1	[0.998 - 1]	0.992	[0.933 - 1]
		1260	1	[1 - 1]	1	[0.998 - 1]	1	[0.998 - 1]	0.994	[0.942 - 1]

		1440	1	[1 - 1]	1	[0.998 - 1]	1	[0.998 - 1]	0.995	[0.981 - 1]
		1620	1	[1 - 1]	1	[0.998 - 1]	1	[0.998 - 1]	0.995	[0.922 - 1]
		1800	1	[1 - 1]	1	[0.998 - 1]	1	[0.998 - 1]	0.995	[0.952 - 1]
	MAF≥5%, Distance≤0.25Mb	180	0.808	[0.556 - 0.922]	0.699	[0.505 - 0.784]	0.724	[0.529 - 0.808]	0.080	[0.008 - 0.224]
		360	1	[0.984 - 1]	0.973	[0.946 - 0.989]	0.979	[0.949 - 0.997]	0.640	[0.288 - 0.832]
		540	1	[1 - 1]	0.997	[0.990 - 1]	0.998	[0.994 - 1]	0.910	[0.527 - 0.978]
		720	1	[1 - 1]	0.998	[0.994 - 1]	1	[0.995 - 1]	0.955	[0.674 - 0.997]
		900	1	[1 - 1]	1	[0.994 - 1]	1	[0.995 - 1]	0.978	[0.780 - 0.998]
		1080	1	[1 - 1]	1	[0.997 - 1]	1	[0.998 - 1]	0.990	[0.834 - 0.998]
		1260	1	[1 - 1]	1	[0.998 - 1]	1	[0.998 - 1]	0.992	[0.837 - 1]
		1440	1	[1 - 1]	1	[0.998 - 1]	1	[0.998 - 1]	0.994	[0.839 - 1]
		1620	1	[1 - 1]	1	[0.998 - 1]	1	[0.998 - 1]	0.995	[0.756 - 1]
		1800	1	[1 - 1]	1	[0.998 - 1]	1	[0.998 - 1]	0.997	[0.915 - 1]
	MAF≥10%, Distance≤0.0625Mb	180	1	[0.992 - 1]	0.982	[0.958 - 0.995]	0.986	[0.971 - 0.997]	0.647	[0.078 - 0.839]
		360	1	[1 - 1]	1	[0.994 - 1]	1	[0.997 - 1]	0.953	[0.818 - 0.989]
		540	1	[1 - 1]	1	[0.995 - 1]	1	[0.997 - 1]	0.978	[0.837 - 0.995]
		720	1	[1 - 1]	1	[0.997 - 1]	1	[0.997 - 1]	0.986	[0.909 - 1]
		900	1	[1 - 1]	1	[0.998 - 1]	1	[0.997 - 1]	0.989	[0.938 - 1]
		1080	1	[1 - 1]	1	[0.997 - 1]	1	[0.997 - 1]	0.990	[0.931 - 1]
		1260	1	[1 - 1]	1	[0.997 - 1]	1	[0.997 - 1]	0.992	[0.954 - 1]
		1440	1	[1 - 1]	1	[0.997 - 1]	1	[0.998 - 1]	0.990	[0.903 - 1]
		1620	1	[1 - 1]	1	[0.998 - 1]	1	[0.998 - 1]	0.992	[0.935 - 1]
		1800	1	[1 - 1]	1	[0.997 - 1]	1	[0.998 - 1]	0.994	[0.930 - 1]
	MAF≥10%, Distance≤0.125Mb	180	0.997	[0.968 - 1]	0.952	[0.863 - 0.986]	0.960	[0.879 - 0.984]	0.389	[0.102 - 0.706]
		360	1	[1 - 1]	0.998	[0.989 - 1]	0.998	[0.994 - 1]	0.908	[0.545 - 0.978]
		540	1	[1 - 1]	1	[0.997 - 1]	1	[0.995 - 1]	0.966	[0.602 - 0.994]

		720	1	[1 - 1]	1	[0.997 - 1]	1	[0.997 - 1]	0.987	[0.788 - 0.998]
		900	1	[1 - 1]	1	[0.998 - 1]	1	[0.998 - 1]	0.992	[0.866 - 1]
		1080	1	[1 - 1]	1	[0.998 - 1]	1	[0.998 - 1]	0.993	[0.952 - 1]
		1260	1	[1 - 1]	1	[0.998 - 1]	1	[0.998 - 1]	0.995	[0.917 - 1]
		1440	1	[1 - 1]	1	[0.998 - 1]	1	[1 - 1]	0.994	[0.968 - 1]
		1620	1	[1 - 1]	1	[0.998 - 1]	1	[0.998 - 1]	0.997	[0.968 - 1]
		1800	1	[1 - 1]	1	[1 - 1]	1	[0.998 - 1]	0.997	[0.952 - 1]
	MAF≥10%, Distance≤0.25Mb	180	0.941	[0.740 - 0.987]	0.830	[0.647 - 0.896]	0.845	[0.658 - 0.899]	0.17	[0.018 - 0.412]
		360	1	[0.997 - 1]	0.987	[0.971 - 0.998]	0.990	[0.978 - 1]	0.749	[0.238 - 0.895]
		540	1	[0.997 - 1]	0.998	[0.987 - 1]	0.998	[0.990 - 1]	0.927	[0.468 - 0.989]
		720	1	[1 - 1]	1	[0.995 - 1]	1	[0.995 - 1]	0.97	[0.542 - 0.998]
		900	1	[1 - 1]	1	[0.998 - 1]	1	[0.997 - 1]	0.986	[0.834 - 1]
		1080	1	[1 - 1]	1	[0.998 - 1]	1	[0.998 - 1]	0.989	[0.732 - 1]
		1260	1	[1 - 1]	1	[0.998 - 1]	1	[0.998 - 1]	0.994	[0.725 - 1]
		1440	1	[1 - 1]	1	[0.997 - 1]	1	[0.998 - 1]	0.995	[0.807 - 1]
		1620	1	[1 - 1]	1	[1 - 1]	1	[0.998 - 1]	0.995	[0.855 - 1]
		1800	1	[1 - 1]	1	[0.998 - 1]	1	[0.998 - 1]	0.996	[0.928 - 1]
	Pop-MAF>0%, Distance≤0.0625Mb	180	1	[0.994 - 1]	0.965	[0.930 - 0.994]	0.974	[0.944 - 0.995]	0.577	[0.157 - 0.869]
		360	1	[1 - 1]	0.998	[0.992 - 1]	0.998	[0.995 - 1]	0.941	[0.757 - 0.984]
		540	1	[1 - 1]	1	[0.997 - 1]	1	[0.997 - 1]	0.977	[0.856 - 0.997]
		720	1	[1 - 1]	1	[0.998 - 1]	1	[0.998 - 1]	0.982	[0.839 - 1]
		900	1	[1 - 1]	1	[0.998 - 1]	1	[0.998 - 1]	0.990	[0.939 - 1]
		1080	1	[1 - 1]	1	[0.998 - 1]	1	[0.998 - 1]	0.992	[0.950 - 1]
		1260	1	[1 - 1]	1	[0.998 - 1]	1	[0.998 - 1]	0.992	[0.952 - 1]
		1440	1	[1 - 1]	1	[0.998 - 1]	1	[0.998 - 1]	0.992	[0.931 - 1]
		1620	1	[1 - 1]	1	[0.998 - 1]	1	[0.998 - 1]	0.994	[0.950 - 1]

		1800	1	[1 - 1]	1	[0.998 - 1]	1	[0.998 - 1]	0.994	[0.954 - 1]
	Pop-MAF>0%, Distance≤0.125Mb	180	0.986	[0.936 - 1]	0.892	[0.804 - 0.952]	0.909	[0.832 - 0.966]	0.238	[0.026 - 0.618]
		360	1	[1 - 1]	0.995	[0.971 - 1]	0.995	[0.978 - 1]	0.854	[0.419 - 0.963]
		540	1	[1 - 1]	1	[0.995 - 1]	1	[0.994 - 1]	0.956	[0.709 - 0.997]
		720	1	[1 - 1]	1	[0.995 - 1]	1	[0.997 - 1]	0.978	[0.816 - 0.997]
		900	1	[1 - 1]	1	[0.998 - 1]	1	[0.997 - 1]	0.990	[0.799 - 1]
		1080	1	[1 - 1]	1	[0.998 - 1]	1	[0.998 - 1]	0.990	[0.930 - 1]
		1260	1	[1 - 1]	1	[0.997 - 1]	1	[0.998 - 1]	0.994	[0.925 - 1]
		1440	1	[1 - 1]	1	[0.998 - 1]	1	[0.998 - 1]	0.995	[0.966 - 1]
		1620	1	[1 - 1]	1	[0.998 - 1]	1	[0.998 - 1]	0.995	[0.971 - 1]
		1800	1	[1 - 1]	1	[0.998 - 1]	1	[0.998 - 1]	0.995	[0.960 - 1]
	Pop-MAF>0%, Distance≤0.25Mb	180	0.759	[0.597 - 0.970]	0.659	[0.519 - 0.840]	0.674	[0.554 - 0.869]	0.063	[0.006 - 0.200]
		360	1	[0.990 - 1]	0.968	[0.915 - 0.987]	0.973	[0.925 - 0.994]	0.558	[0.126 - 0.824]
		540	1	[1 - 1]	0.997	[0.987 - 1]	0.998	[0.992 - 1]	0.889	[0.486 - 0.965]
		720	1	[1 - 1]	0.998	[0.990 - 1]	1	[0.994 - 1]	0.946	[0.537 - 0.990]
		900	1	[1 - 1]	1	[0.995 - 1]	1	[0.995 - 1]	0.970	[0.720 - 1]
		1080	1	[1 - 1]	1	[0.998 - 1]	1	[0.997 - 1]	0.987	[0.607 - 1]
		1260	1	[1 - 1]	1	[0.998 - 1]	1	[0.998 - 1]	0.992	[0.757 - 1]
		1440	1	[1 - 1]	1	[0.997 - 1]	1	[0.998 - 1]	0.994	[0.927 - 1]
		1620	1	[1 - 1]	1	[0.998 - 1]	1	[0.998 - 1]	0.995	[0.839 - 1]
		1800	1	[1 - 1]	1	[0.998 - 1]	1	[0.998 - 1]	0.995	[0.835 - 1]
	Pop-MAF≥1%, Distance≤0.0625Mb	180	1	[0.995 - 1]	0.979	[0.949 - 0.995]	0.982	[0.960 - 0.998]	0.62	[0.115 - 0.853]
		360	1	[1 - 1]	0.998	[0.994 - 1]	1	[0.994 - 1]	0.95	[0.270 - 0.989]
		540	1	[1 - 1]	1	[0.997 - 1]	1	[0.997 - 1]	0.981	[0.767 - 1]
		720	1	[1 - 1]	1	[0.997 - 1]	1	[0.998 - 1]	0.984	[0.895 - 1]
		900	1	[1 - 1]	1	[0.997 - 1]	1	[0.998 - 1]	0.992	[0.936 - 1]

		1080	1	[1 - 1]	1	[0.997 - 1]	1	[0.997 - 1]	0.994	[0.954 - 1]
		1260	1	[1 - 1]	1	[0.998 - 1]	1	[0.997 - 1]	0.995	[0.947 - 1]
		1440	1	[1 - 1]	1	[0.998 - 1]	1	[0.998 - 1]	0.994	[0.950 - 1]
		1620	1	[1 - 1]	1	[0.998 - 1]	1	[0.998 - 1]	0.995	[0.935 - 1]
		1800	1	[1 - 1]	1	[0.998 - 1]	1	[0.998 - 1]	0.994	[0.936 - 1]
	Pop-MAF≥1%, Distance≤0.125Mb	180	0.997	[0.970 - 1]	0.928	[0.885 - 0.962]	0.942	[0.899 - 0.974]	0.371	[0.032 - 0.615]
		360	1	[1 - 1]	0.997	[0.989 - 1]	0.998	[0.990 - 1]	0.899	[0.452 - 0.976]
		540	1	[1 - 1]	1	[0.995 - 1]	1	[0.997 - 1]	0.971	[0.682 - 0.994]
		720	1	[1 - 1]	1	[0.997 - 1]	1	[0.998 - 1]	0.987	[0.879 - 1]
		900	1	[1 - 1]	1	[0.997 - 1]	1	[0.998 - 1]	0.989	[0.880 - 1]
		1080	1	[1 - 1]	1	[0.998 - 1]	1	[0.998 - 1]	0.992	[0.946 - 1]
		1260	1	[1 - 1]	1	[0.998 - 1]	1	[0.998 - 1]	0.994	[0.963 - 1]
		1440	1	[1 - 1]	1	[0.998 - 1]	1	[0.998 - 1]	0.994	[0.962 - 1]
		1620	1	[1 - 1]	1	[0.998 - 1]	1	[0.998 - 1]	0.995	[0.966 - 1]
		1800	1	[1 - 1]	1	[0.998 - 1]	1	[0.998 - 1]	0.995	[0.974 - 1]
	Pop-MAF≥1%, Distance≤0.25Mb	180	0.884	[0.743 - 0.978]	0.773	[0.652 - 0.875]	0.789	[0.652 - 0.882]	0.112	[0.019 - 0.312]
		360	1	[0.990 - 1]	0.987	[0.955 - 0.998]	0.989	[0.973 - 1]	0.733	[0.355 - 0.925]
		540	1	[1 - 1]	0.997	[0.990 - 1]	0.998	[0.992 - 1]	0.907	[0.516 - 0.982]
		720	1	[1 - 1]	1	[0.994 - 1]	1	[0.995 - 1]	0.958	[0.617 - 0.992]
		900	1	[1 - 1]	1	[0.997 - 1]	1	[0.997 - 1]	0.979	[0.500 - 1]
		1080	1	[1 - 1]	1	[0.997 - 1]	1	[0.998 - 1]	0.990	[0.829 - 1]
		1260	1	[1 - 1]	1	[0.998 - 1]	1	[0.998 - 1]	0.994	[0.856 - 1]
		1440	1	[1 - 1]	1	[0.998 - 1]	1	[0.998 - 1]	0.994	[0.855 - 1]
		1620	1	[1 - 1]	1	[0.998 - 1]	1	[0.998 - 1]	0.995	[0.719 - 1]
		1800	1	[1 - 1]	1	[0.998 - 1]	1	[1 - 1]	0.997	[0.923 - 1]
	Pop-MAF≥5%, Distance≤0.0625Mb	180	1	[0.995 - 1]	0.984	[0.960 - 0.998]	0.988	[0.965 - 1]	0.702	[0.249 - 0.898]

		360	1	[1 - 1]	1	[0.992 - 1]	1	[0.997 - 1]	0.942	[0.780 - 0.995]
		540	1	[1 - 1]	1	[0.997 - 1]	1	[0.998 - 1]	0.981	[0.895 - 0.998]
		720	1	[1 - 1]	1	[0.997 - 1]	1	[0.998 - 1]	0.985	[0.911 - 0.998]
		900	1	[1 - 1]	1	[0.998 - 1]	1	[0.997 - 1]	0.987	[0.901 - 1]
		1080	1	[1 - 1]	1	[0.998 - 1]	1	[0.998 - 1]	0.990	[0.890 - 0.998]
		1260	1	[1 - 1]	1	[0.998 - 1]	1	[0.998 - 1]	0.989	[0.904 - 1]
		1440	1	[1 - 1]	1	[0.997 - 1]	1	[0.998 - 1]	0.992	[0.920 - 1]
		1620	1	[1 - 1]	1	[0.995 - 1]	1	[0.997 - 1]	0.992	[0.911 - 1]
		1800	1	[1 - 1]	1	[0.997 - 1]	1	[0.997 - 1]	0.994	[0.919 - 1]
	Pop-MAF≥5%, Distance≤0.125Mb	180	1	[0.982 - 1]	0.951	[0.909 - 0.99]	0.962	[0.927 - 0.989]	0.458	[0.166 - 0.749]
		360	1	[1 - 1]	0.998	[0.994 - 1]	0.998	[0.992 - 1]	0.930	[0.516 - 0.987]
		540	1	[1 - 1]	1	[0.997 - 1]	1	[0.998 - 1]	0.978	[0.823 - 0.998]
		720	1	[1 - 1]	1	[0.998 - 1]	1	[0.997 - 1]	0.989	[0.906 - 0.998]
		900	1	[1 - 1]	1	[0.998 - 1]	1	[0.998 - 1]	0.994	[0.949 - 1]
		1080	1	[1 - 1]	1	[0.998 - 1]	1	[0.998 - 1]	0.992	[0.887 - 1]
		1260	1	[1 - 1]	1	[0.998 - 1]	1	[0.998 - 1]	0.994	[0.970 - 1]
		1440	1	[1 - 1]	1	[1 - 1]	1	[0.998 - 1]	0.994	[0.962 - 1]
		1620	1	[1 - 1]	1	[0.998 - 1]	1	[0.997 - 1]	0.995	[0.971 - 1]
		1800	1	[1 - 1]	1	[0.998 - 1]	1	[0.997 - 1]	0.995	[0.968 - 1]
	Pop-MAF≥5%, Distance≤0.25Mb	180	0.904	[0.778 - 0.966]	0.792	[0.692 - 0.855]	0.804	[0.687 - 0.867]	0.145	[0.021 - 0.307]
		360	1	[0.994 - 1]	0.990	[0.968 - 0.998]	0.990	[0.971 - 1]	0.726	[0.363 - 0.917]
		540	1	[1 - 1]	0.998	[0.994 - 1]	0.998	[0.992 - 1]	0.933	[0.668 - 0.987]
		720	1	[1 - 1]	1	[0.997 - 1]	1	[0.997 - 1]	0.974	[0.604 - 0.995]
		900	1	[1 - 1]	1	[0.998 - 1]	1	[0.998 - 1]	0.987	[0.668 - 1]
		1080	1	[1 - 1]	1	[0.997 - 1]	1	[0.998 - 1]	0.990	[0.808 - 1]
		1260	1	[1 - 1]	1	[0.998 - 1]	1	[0.998 - 1]	0.994	[0.677 - 1]

		1440	1	[1 - 1]	1	[0.998 - 1]	1	[0.998 - 1]	0.995	[0.756 - 1]
		1620	1	[1 - 1]	1	[1 - 1]	1	[0.998 - 1]	0.995	[0.700 - 1]
		1800	1	[1 - 1]	1	[0.998 - 1]	1	[0.998 - 1]	0.997	[0.741 - 1]
**B. MAF or pop-MAF, distance, and *D'*$_{avg}$ conditions (*n*=16 combinations with at least 100 SNPs per CODIS STR)**										
	Distance≤0.0625Mb, D'_{avg}≥0.3	180	0.617	[0.428 - 0.834]	0.430	[0.220 - 0.631]	0.554	[0.407 - 0.749]	0.030	[0 - 0.131]
		360	0.979	[0.864 - 1]	0.852	[0.696 - 0.931]	0.897	[0.767 - 0.957]	0.258	[0.013 - 0.524]
		540	1	[0.995 - 1]	0.965	[0.930 - 0.990]	0.979	[0.950 - 0.997]	0.595	[0.161 - 0.869]
		720	1	[0.997 - 1]	0.990	[0.966 - 1]	0.994	[0.974 - 1]	0.785	[0.304 - 0.949]
		900	1	[0.994 - 1]	0.996	[0.981 - 1]	0.997	[0.986 - 1]	0.865	[0.385 - 0.970]
		1080	1	[1 - 1]	0.998	[0.990 - 1]	0.998	[0.994 - 1]	0.92	[0.281 - 0.978]
		1260	1	[1 - 1]	0.998	[0.992 - 1]	0.998	[0.994 - 1]	0.925	[0.601 - 0.981]
		1440	1	[1 - 1]	0.998	[0.994 - 1]	1	[0.995 - 1]	0.957	[0.681 - 0.990]
		1620	1	[1 - 1]	1	[0.994 - 1]	1	[0.997 - 1]	0.962	[0.725 - 1]
		1800	1	[1 - 1]	1	[0.995 - 1]	1	[0.997 - 1]	0.974	[0.794 - 0.995]
	Distance≤0.0625Mb, D'_{avg}≥0.5	180	0.499	[0.256 - 0.858]	0.305	[0.142 - 0.602]	0.466	[0.238 - 0.760]	0.019	[0 - 0.136]
		360	0.950	[0.842 - 0.994]	0.766	[0.617 - 0.872]	0.860	[0.768 - 0.938]	0.188	[0.005 - 0.382]
		540	0.998	[0.978 - 1]	0.927	[0.879 - 0.974]	0.962	[0.928 - 0.990]	0.452	[0.125 - 0.733]
		720	1	[0.992 - 1]	0.971	[0.946 - 0.990]	0.987	[0.965 - 0.995]	0.631	[0.351 - 0.845]
		900	1	[0.997 - 1]	0.986	[0.941 - 1]	0.992	[0.971 - 1]	0.728	[0.318 - 0.939]
		1080	1	[0.997 - 1]	0.994	[0.978 - 1]	0.997	[0.984 - 1]	0.828	[0.307 - 0.958]
		1260	1	[1 - 1]	0.995	[0.984 - 1]	0.998	[0.989 - 1]	0.866	[0.519 - 0.984]
		1440	1	[0.997 - 1]	0.997	[0.989 - 1]	0.998	[0.987 - 1]	0.910	[0.690 - 0.992]
		1620	1	[1 - 1]	0.998	[0.990 - 1]	0.998	[0.994 - 1]	0.927	[0.701 - 0.981]
		1800	1	[1 - 1]	0.998	[0.995 - 1]	1	[0.995 - 1]	0.949	[0.637 - 0.989]
	Distance≤0.0625Mb, D'_{avg}≥0.7	180	0.446	[0.192 - 0.762]	0.230	[0.102 - 0.505]	0.406	[0.208 - 0.692]	0.014	[0 - 0.070]
		360	0.843	[0.644 - 0.971]	0.597	[0.417 - 0.796]	0.756	[0.594 - 0.887]	0.072	[0.003 - 0.244]

		540	0.960	[0.855 - 1]	0.774	[0.596 - 0.898]	0.879	[0.746 - 0.958]	0.187	[0.038 - 0.340]
		720	0.996	[0.947 - 1]	0.907	[0.780 - 0.966]	0.96	[0.888 - 0.989]	0.382	[0.171 - 0.693]
		900	0.997	[0.974 - 1]	0.930	[0.821 - 0.978]	0.965	[0.914 - 0.994]	0.450	[0.096 - 0.698]
		1080	1	[0.994 - 1]	0.973	[0.930 - 0.995]	0.986	[0.963 - 0.998]	0.621	[0.134 - 0.879]
		1260	1	[0.994 - 1]	0.981	[0.933 - 0.995]	0.992	[0.965 - 1]	0.704	[0.200 - 0.898]
		1440	1	[0.994 - 1]	0.982	[0.954 - 0.998]	0.994	[0.982 - 0.998]	0.740	[0.256 - 0.898]
		1620	1	[0.997 - 1]	0.992	[0.976 - 1]	0.997	[0.989 - 1]	0.804	[0.564 - 0.968]
		1800	1	[0.997 - 1]	0.994	[0.982 - 1]	0.998	[0.989 - 1]	0.823	[0.532 - 0.955]
	Distance≤0.125Mb, D'_{avg}≥0.3	180	0.345	[0.190 - 0.519]	0.238	[0.107 - 0.356]	0.334	[0.206 - 0.458]	0.011	[0 - 0.042]
		360	0.841	[0.583 - 0.979]	0.669	[0.427 - 0.853]	0.754	[0.542 - 0.899]	0.112	[0.002 - 0.395]
		540	0.984	[0.871 - 1]	0.879	[0.703 - 0.950]	0.911	[0.791 - 0.966]	0.331	[0.070 - 0.644]
		720	0.997	[0.955 - 1]	0.939	[0.887 - 0.989]	0.958	[0.906 - 0.997]	0.495	[0.136 - 0.796]
		900	1	[0.987 - 1]	0.979	[0.933 - 0.995]	0.986	[0.949 - 0.997]	0.689	[0.192 - 0.875]
		1080	1	[0.995 - 1]	0.986	[0.957 - 1]	0.990	[0.965 - 1]	0.745	[0.230 - 0.903]
		1260	1	[0.997 - 1]	0.994	[0.978 - 1]	0.995	[0.982 - 1]	0.851	[0.481 - 0.981]
		1440	1	[1 - 1]	0.997	[0.989 - 1]	0.998	[0.989 - 1]	0.919	[0.450 - 0.978]
		1620	1	[1 - 1]	0.998	[0.990 - 1]	0.998	[0.990 - 1]	0.922	[0.340 - 0.990]
		1800	1	[1 - 1]	0.998	[0.992 - 1]	0.998	[0.994 - 1]	0.947	[0.564 - 0.992]
	Distance≤0.125Mb, D'_{avg}≥0.5	180	0.383	[0.153 - 0.577]	0.242	[0.072 - 0.361]	0.367	[0.158 - 0.534]	0.013	[0 - 0.046]
		360	0.737	[0.454 - 0.907]	0.518	[0.321 - 0.724]	0.663	[0.436 - 0.813]	0.073	[0.008 - 0.184]
		540	0.965	[0.634 - 1]	0.833	[0.476 - 0.912]	0.895	[0.593 - 0.955]	0.216	[0.014 - 0.430]
		720	0.994	[0.903 - 1]	0.911	[0.765 - 0.976]	0.945	[0.834 - 0.989]	0.374	[0.093 - 0.684]
		900	1	[0.986 - 1]	0.950	[0.922 - 0.992]	0.970	[0.949 - 0.995]	0.542	[0.160 - 0.784]
		1080	1	[0.994 - 1]	0.978	[0.947 - 0.994]	0.987	[0.963 - 0.998]	0.661	[0.351 - 0.843]
		1260	1	[0.997 - 1]	0.984	[0.963 - 0.998]	0.990	[0.974 - 0.998]	0.739	[0.147 - 0.939]
		1440	1	[0.997 - 1]	0.994	[0.971 - 1]	0.997	[0.981 - 1]	0.839	[0.527 - 0.966]

		1620	1	[0.997 - 1]	0.995	[0.986 - 1]	0.998	[0.990 - 1]	0.870	[0.272 - 0.955]
		1800	1	[0.997 - 1]	0.997	[0.982 - 1]	0.998	[0.986 - 1]	0.866	[0.435 - 0.976]
	Distance≤0.125Mb, $D'_{avg} \geq 0.7$	180	0.288	[0.083 - 0.412]	0.157	[0.050 - 0.248]	0.279	[0.105 - 0.393]	0.006	[0 - 0.026]
		360	0.633	[0.308 - 0.794]	0.415	[0.174 - 0.562]	0.577	[0.292 - 0.724]	0.042	[0.005 - 0.128]
		540	0.866	[0.471 - 0.986]	0.654	[0.319 - 0.855]	0.788	[0.412 - 0.928]	0.101	[0.006 - 0.347]
		720	0.946	[0.740 - 1]	0.784	[0.550 - 0.896]	0.874	[0.698 - 0.946]	0.191	[0.069 - 0.403]
		900	0.990	[0.879 - 1]	0.882	[0.733 - 0.957]	0.935	[0.837 - 0.982]	0.357	[0.102 - 0.631]
		1080	0.994	[0.954 - 1]	0.908	[0.796 - 0.971]	0.954	[0.879 - 0.984]	0.411	[0.137 - 0.786]
		1260	1	[0.974 - 1]	0.944	[0.896 - 0.984]	0.971	[0.931 - 0.994]	0.490	[0.157 - 0.791]
		1440	1	[0.989 - 1]	0.973	[0.947 - 0.989]	0.986	[0.968 - 0.997]	0.658	[0.292 - 0.864]
		1620	1	[0.990 - 1]	0.979	[0.942 - 0.992]	0.989	[0.971 - 0.997]	0.685	[0.169 - 0.853]
		1800	1	[0.997 - 1]	0.982	[0.957 - 0.997]	0.990	[0.978 - 0.998]	0.676	[0.347 - 0.885]
	Distance≤0.25Mb, $D'_{avg} \geq 0.3$	180	0.177	[0.062 - 0.334]	0.122	[0.037 - 0.236]	0.185	[0.061 - 0.327]	0.003	[0 - 0.019]
		360	0.553	[0.428 - 0.781]	0.426	[0.313 - 0.615]	0.511	[0.380 - 0.684]	0.035	[0.003 - 0.147]
		540	0.856	[0.478 - 0.957]	0.712	[0.398 - 0.834]	0.772	[0.449 - 0.872]	0.118	[0.021 - 0.268]
		720	0.954	[0.796 - 1]	0.835	[0.696 - 0.949]	0.867	[0.741 - 0.963]	0.257	[0.048 - 0.561]
		900	0.982	[0.906 - 1]	0.881	[0.797 - 0.979]	0.912	[0.839 - 0.981]	0.359	[0.089 - 0.645]
		1080	0.997	[0.960 - 1]	0.947	[0.882 - 0.986]	0.958	[0.909 - 0.992]	0.526	[0.128 - 0.760]
		1260	1	[0.981 - 1]	0.978	[0.939 - 0.997]	0.982	[0.949 - 0.998]	0.629	[0.268 - 0.859]
		1440	1	[0.994 - 1]	0.981	[0.949 - 1]	0.986	[0.965 - 1]	0.687	[0.254 - 0.930]
		1620	1	[0.997 - 1]	0.989	[0.960 - 1]	0.992	[0.968 - 0.998]	0.748	[0.423 - 0.931]
		1800	1	[0.995 - 1]	0.989	[0.963 - 0.998]	0.992	[0.968 - 0.998]	0.796	[0.412 - 0.933]
	Distance≤0.25Mb, $D'_{avg} \geq 0.5$	180	0.112	[0.040 - 0.252]	0.065	[0.019 - 0.150]	0.128	[0.046 - 0.227]	0.002	[0 - 0.018]
		360	0.514	[0.327 - 0.794]	0.373	[0.200 - 0.591]	0.479	[0.315 - 0.711]	0.029	[0.003 - 0.128]
		540	0.710	[0.436 - 0.984]	0.538	[0.318 - 0.842]	0.643	[0.401 - 0.896]	0.062	[0.010 - 0.262]
		720	0.918	[0.719 - 0.997]	0.764	[0.562 - 0.919]	0.827	[0.650 - 0.941]	0.181	[0.050 - 0.414]

		900	0.982	[0.864 - 1]	0.882	[0.732 - 0.942]	0.915	[0.799 - 0.957]	0.308	[0.091 - 0.538]
		1080	0.997	[0.904 - 1]	0.944	[0.780 - 0.982]	0.960	[0.832 - 0.987]	0.451	[0.163 - 0.756]
		1260	1	[0.979 - 1]	0.954	[0.883 - 0.992]	0.966	[0.915 - 0.995]	0.528	[0.147 - 0.840]
		1440	0.998	[0.968 - 1]	0.960	[0.901 - 0.987]	0.968	[0.917 - 0.992]	0.554	[0.088 - 0.799]
		1620	1	[0.987 - 1]	0.973	[0.933 - 0.994]	0.979	[0.955 - 0.997]	0.618	[0.228 - 0.834]
		1800	1	[0.994 - 1]	0.982	[0.954 - 0.998]	0.987	[0.963 - 0.998]	0.701	[0.409 - 0.906]
		180	0.062	[0.016 - 0.188]	0.041	[0.006 - 0.113]	0.083	[0.035 - 0.211]	0.002	[0 - 0.016]
		360	0.327	[0.169 - 0.650]	0.211	[0.096 - 0.414]	0.308	[0.184 - 0.602]	0.017	[0.002 - 0.053]
		540	0.675	[0.436 - 0.955]	0.481	[0.292 - 0.770]	0.609	[0.435 - 0.864]	0.066	[0.008 - 0.241]
		720	0.787	[0.604 - 0.984]	0.601	[0.447 - 0.859]	0.715	[0.540 - 0.914]	0.094	[0.006 - 0.312]
	Distance≤0.25Mb, D'_{avg}≥0.7	900	0.899	[0.692 - 1]	0.744	[0.529 - 0.872]	0.823	[0.641 - 0.915]	0.178	[0.050 - 0.377]
		1080	0.955	[0.816 - 1]	0.826	[0.653 - 0.965]	0.883	[0.741 - 0.981]	0.244	[0.046 - 0.589]
		1260	0.985	[0.915 - 1]	0.882	[0.775 - 0.947]	0.924	[0.837 - 0.974]	0.296	[0.088 - 0.569]
		1440	0.997	[0.931 - 1]	0.941	[0.799 - 0.992]	0.957	[0.867 - 0.997]	0.447	[0.067 - 0.772]
		1620	0.997	[0.978 - 1]	0.944	[0.864 - 0.986]	0.963	[0.915 - 0.990]	0.482	[0.165 - 0.776]
		1800	0.998	[0.982 - 1]	0.957	[0.907 - 0.986]	0.971	[0.939 - 0.992]	0.557	[0.273 - 0.773]
		180	0.613	[0.423 - 0.847]	0.482	[0.321 - 0.682]	0.558	[0.396 - 0.762]	0.035	[0.002 - 0.163]
		360	0.987	[0.935 - 1]	0.895	[0.812 - 0.958]	0.923	[0.856 - 0.981]	0.324	[0.088 - 0.588]
		540	1	[0.960 - 1]	0.963	[0.887 - 0.992]	0.973	[0.911 - 0.995]	0.599	[0.273 - 0.872]
		720	1	[0.997 - 1]	0.989	[0.973 - 1]	0.990	[0.976 - 1]	0.782	[0.374 - 0.949]
	MAF≥1%, D'_{avg}≥0.3	900	1	[0.997 - 1]	0.995	[0.981 - 1]	0.997	[0.981 - 1]	0.877	[0.538 - 0.963]
		1080	1	[1 - 1]	0.998	[0.994 - 1]	0.998	[0.994 - 1]	0.939	[0.749 - 0.987]
		1260	1	[1 - 1]	0.998	[0.995 - 1]	0.999	[0.995 - 1]	0.951	[0.639 - 0.987]
		1440	1	[1 - 1]	1	[0.997 - 1]	1	[0.997 - 1]	0.973	[0.834 - 0.998]
		1620	1	[1 - 1]	1	[0.997 - 1]	1	[0.997 - 1]	0.979	[0.794 - 1]
		1800	1	[1 - 1]	1	[0.997 - 1]	1	[0.998 - 1]	0.986	[0.796 - 0.998]

	MAF≥1%, D'_{avg}≥0.5	180	0.970	[0.807 - 1]	0.828	[0.597 - 0.936]	0.887	[0.714 - 0.965]	0.201	[0.014 - 0.479]
		360	1	[0.995 - 1]	0.982	[0.952 - 0.998]	0.989	[0.971 - 0.998]	0.707	[0.292 - 0.855]
		540	1	[0.997 - 1]	0.995	[0.987 - 1]	0.997	[0.989 - 1]	0.861	[0.636 - 0.957]
		720	1	[1 - 1]	0.998	[0.990 - 1]	0.998	[0.992 - 1]	0.932	[0.661 - 0.984]
		900	1	[1 - 1]	0.998	[0.992 - 1]	1	[0.995 - 1]	0.957	[0.786 - 0.992]
		1080	1	[1 - 1]	1	[0.997 - 1]	1	[0.997 - 1]	0.971	[0.770 - 0.997]
		1260	1	[1 - 1]	1	[0.995 - 1]	1	[0.997 - 1]	0.978	[0.700 - 0.997]
		1440	1	[1 - 1]	1	[0.997 - 1]	1	[0.997 - 1]	0.983	[0.843 - 0.998]
		1620	1	[1 - 1]	1	[0.997 - 1]	1	[0.998 - 1]	0.986	[0.856 - 0.998]
		1800	1	[1 - 1]	1	[0.995 - 1]	1	[0.997 - 1]	0.990	[0.829 - 0.998]
	MAF≥1%, Distance≤0.0625Mb, D'_{avg}≥0.3	180	0.994	[0.923 - 1]	0.904	[0.781 - 0.976]	0.935	[0.842 - 0.986]	0.342	[0.072 - 0.751]
		360	1	[0.997 - 1]	0.994	[0.978 - 1]	0.995	[0.984 - 1]	0.825	[0.481 - 0.941]
		540	1	[1 - 1]	0.998	[0.994 - 1]	1	[0.997 - 1]	0.944	[0.744 - 0.989]
		720	1	[1 - 1]	1	[0.995 - 1]	1	[0.997 - 1]	0.966	[0.698 - 0.998]
		900	1	[1 - 1]	1	[0.997 - 1]	1	[0.998 - 1]	0.978	[0.864 - 1]
		1080	1	[1 - 1]	1	[0.997 - 1]	1	[0.995 - 1]	0.984	[0.840 - 1]
		1260	1	[1 - 1]	1	[0.997 - 1]	1	[0.998 - 1]	0.986	[0.867 - 1]
		1440	1	[1 - 1]	1	[0.998 - 1]	1	[0.997 - 1]	0.987	[0.831 - 1]
		1620	1	[1 - 1]	1	[0.997 - 1]	1	[0.998 - 1]	0.992	[0.887 - 1]
		1800	1	[1 - 1]	1	[0.998 - 1]	1	[0.998 - 1]	0.992	[0.930 - 1]
	MAF≥1%, Distance≤0.125Mb, D'_{avg}≥0.3	180	0.935	[0.826 - 1]	0.782	[0.652 - 0.917]	0.841	[0.727 - 0.939]	0.193	[0.013 - 0.470]
		360	1	[0.994 - 1]	0.981	[0.957 - 0.997]	0.987	[0.966 - 1]	0.730	[0.211 - 0.895]
		540	1	[1 - 1]	0.997	[0.992 - 1]	0.998	[0.990 - 1]	0.909	[0.457 - 0.992]
		720	1	[1 - 1]	0.998	[0.994 - 1]	1	[0.995 - 1]	0.952	[0.695 - 0.989]
		900	1	[1 - 1]	1	[0.997 - 1]	1	[0.997 - 1]	0.974	[0.706 - 0.997]
		1080	1	[1 - 1]	1	[0.997 - 1]	1	[0.997 - 1]	0.979	[0.891 - 0.998]

		1260	1	[1 - 1]	1	[0.998 - 1]	1	[0.998 - 1]	0.986	[0.933 - 1]
		1440	1	[1 - 1]	1	[0.997 - 1]	1	[0.998 - 1]	0.990	[0.864 - 1]
		1620	1	[1 - 1]	1	[0.998 - 1]	1	[0.998 - 1]	0.993	[0.962 - 1]
		1800	1	[1 - 1]	1	[0.998 - 1]	1	[0.998 - 1]	0.994	[0.931 - 1]
	MAF≥1%, Distance≤0.125Mb, D'_{avg}≥0.5	180	0.987	[0.930 - 1]	0.859	[0.781 - 0.954]	0.918	[0.847 - 0.965]	0.305	[0.038 - 0.610]
		360	1	[0.994 - 1]	0.990	[0.968 - 0.998]	0.995	[0.971 - 1]	0.796	[0.444 - 0.944]
		540	1	[0.997 - 1]	0.997	[0.989 - 1]	0.998	[0.994 - 1]	0.894	[0.633 - 0.974]
		720	1	[1 - 1]	1	[0.992 - 1]	1	[0.995 - 1]	0.955	[0.513 - 0.989]
		900	1	[1 - 1]	1	[0.997 - 1]	1	[0.998 - 1]	0.968	[0.692 - 0.995]
		1080	1	[1 - 1]	1	[0.997 - 1]	1	[0.994 - 1]	0.973	[0.773 - 0.995]
		1260	1	[1 - 1]	1	[0.995 - 1]	1	[0.998 - 1]	0.982	[0.837 - 0.995]
		1440	1	[1 - 1]	1	[0.997 - 1]	1	[0.998 - 1]	0.986	[0.885 - 1]
		1620	1	[1 - 1]	1	[0.997 - 1]	1	[0.997 - 1]	0.986	[0.840 - 0.998]
		1800	1	[1 - 1]	1	[0.997 - 1]	1	[0.997 - 1]	0.986	[0.898 - 0.998]
	MAF≥1%, Distance≤0.25Mb, D'_{avg}≥0.3	180	0.831	[0.604 - 0.963]	0.664	[0.470 - 0.821]	0.740	[0.565 - 0.867]	0.088	[0.011 - 0.268]
		360	1	[0.981 - 1]	0.963	[0.914 - 0.994]	0.971	[0.939 - 0.995]	0.478	[0.075 - 0.863]
		540	1	[0.997 - 1]	0.990	[0.974 - 0.998]	0.992	[0.981 - 1]	0.796	[0.487 - 0.939]
		720	1	[1 - 1]	0.998	[0.992 - 1]	0.998	[0.994 - 1]	0.918	[0.505 - 0.981]
		900	1	[1 - 1]	1	[0.994 - 1]	1	[0.995 - 1]	0.958	[0.641 - 0.995]
		1080	1	[1 - 1]	1	[0.994 - 1]	1	[0.995 - 1]	0.966	[0.711 - 0.997]
		1260	1	[1 - 1]	1	[0.995 - 1]	1	[0.997 - 1]	0.981	[0.647 - 1]
		1440	1	[1 - 1]	1	[0.998 - 1]	1	[0.998 - 1]	0.985	[0.901 - 1]
		1620	1	[1 - 1]	1	[0.998 - 1]	1	[0.998 - 1]	0.987	[0.931 - 1]
		1800	1	[1 - 1]	1	[0.998 - 1]	1	[0.998 - 1]	0.992	[0.850 - 1]
	MAF≥1%, Distance≤0.25Mb, D'_{avg}≥0.5	180	0.965	[0.816 - 1]	0.814	[0.593 - 0.904]	0.884	[0.749 - 0.944]	0.211	[0.061 - 0.427]
		360	1	[0.995 - 1]	0.986	[0.957 - 0.998]	0.992	[0.978 - 0.998]	0.731	[0.332 - 0.914]

		540	1	[0.997 - 1]	0.995	[0.986 - 1]	0.998	[0.990 - 1]	0.872	[0.294 - 0.963]
		720	1	[1 - 1]	0.998	[0.990 - 1]	1	[0.994 - 1]	0.941	[0.701 - 0.984]
		900	1	[1 - 1]	1	[0.994 - 1]	1	[0.995 - 1]	0.963	[0.652 - 0.994]
		1080	1	[1 - 1]	1	[0.995 - 1]	1	[0.997 - 1]	0.971	[0.754 - 0.995]
		1260	1	[1 - 1]	1	[0.997 - 1]	1	[0.998 - 1]	0.979	[0.764 - 0.998]
		1440	1	[0.997 - 1]	1	[0.997 - 1]	1	[0.998 - 1]	0.981	[0.676 - 0.998]
		1620	1	[1 - 1]	1	[0.997 - 1]	1	[0.997 - 1]	0.987	[0.807 - 1]
		1800	1	[1 - 1]	1	[0.997 - 1]	1	[0.998 - 1]	0.982	[0.867 - 1]

Table S6. Record-matching accuracies for sets of SNPs that possess two or more specific characteristics. (A) MAF or pop-MAF and distance conditions, (B) MAF or pop-MAF, distance, and D'_{avg} conditions. Rows highlighted in light pink correspond to SNP sets with matching accuracies comparable to those produced by the full set of 192,672 SNPs: a median=1 for one-to-one, SNP query and STR query, and a median≥0.99 for needle-in-haystack. Table (A) shows medians and ranges plotted in Figure 3. Table (B) shows medians and ranges plotted in Figure S2.

			One-to-one		SNP query		STR query		Needle-in-haystack	
**Condition**	**SNP panel size**	**Super-population**	**Median**	**Range**	**Median**	**Range**	**Median**	**Range**	**Median**	**Range**
MAF≥5%, Distance≤0.125Mb	180	AFR	0.992	[0.941 - 1]	0.914	[0.817 - 0.975]	0.929	[0.839 - 0.986]	0.485	[0.160 - 0.732]
		AMR	0.992	[0.943 - 1]	0.930	[0.859 - 1]	0.942	[0.871 - 1]	0.306	[0.059 - 0.578]
		EAS	0.983	[0.875 - 1]	0.898	[0.750 - 0.984]	0.918	[0.773 - 1]	0.279	[0.041 - 0.593]
		EUR	0.987	[0.934 - 1]	0.905	[0.806 - 0.985]	0.919	[0.838 - 0.976]	0.251	[0.031 - 0.485]
		SAS	0.983	[0.913 - 1]	0.885	[0.773 - 0.969]	0.902	[0.796 - 0.976]	0.228	[0 - 0.545]
	360	AFR	1	[1 - 1]	0.999	[0.988 - 1]	0.999	[0.990 - 1]	0.952	[0.741 - 1]
		AMR	1	[1 - 1]	0.997	[0.976 - 1]	0.998	[0.976 - 1]	0.864	[0.446 - 0.989]
		EAS	1	[0.984 - 1]	0.995	[0.961 - 1]	0.995	[0.969 - 1]	0.836	[0.420 - 1]
		EUR	1	[0.986 - 1]	0.992	[0.969 - 1]	0.994	[0.974 - 1]	0.783	[0.411 - 0.961]
		SAS	1	[1 - 1]	0.992	[0.968 - 1]	0.994	[0.970 - 1]	0.762	[0.320 - 0.976]
	540	AFR	1	[1 - 1]	1	[1 - 1]	1	[1 - 1]	0.992	[0.919 - 1]
		AMR	1	[1 - 1]	1	[0.988 - 1]	1	[0.988 - 1]	0.959	[0.678 - 1]
		EAS	1	[1 - 1]	0.998	[0.981 - 1]	0.999	[0.984 - 1]	0.947	[0.656 - 1]
		EUR	1	[1 - 1]	0.998	[0.984 - 1]	0.999	[0.985 - 1]	0.919	[0.589 - 1]
		SAS	1	[1 - 1]	0.999	[0.983 - 1]	0.999	[0.990 - 1]	0.900	[0.598 - 0.992]
	720	AFR	1	[1 - 1]	1	[0.994 - 1]	1	[1 - 1]	0.994	[0.943 - 1]
		AMR	1	[1 - 1]	1	[0.988 - 1]	1	[0.989 - 1]	0.972	[0.747 - 1]
		EAS	1	[1 - 1]	0.999	[0.991 - 1]	1	[0.991 - 1]	0.964	[0.718 - 1]
		EUR	1	[1 - 1]	0.999	[0.984 - 1]	1	[0.991 - 1]	0.945	[0.637 - 1]
		SAS	1	[1 - 1]	0.999	[0.990 - 1]	0.999	[0.990 - 1]	0.935	[0.661 - 0.992]
	900	AFR	1	[1 - 1]	1	[1 - 1]	1	[0.995 - 1]	0.997	[0.977 - 1]
		AMR	1	[1 - 1]	1	[0.988 - 1]	1	[1 - 1]	0.989	[0.943 - 1]

		EAS	1	[1 - 1]	1	[0.992 - 1]	1	[0.992 - 1]	0.987	[0.824 - 1]
		EUR	1	[1 - 1]	1	[0.992 - 1]	1	[0.992 - 1]	0.976	[0.823 - 1]
		SAS	1	[1 - 1]	1	[0.992 - 1]	1	[0.990 - 1]	0.971	[0.786 - 1]
	1080	AFR	1	[1 - 1]	1	[1 - 1]	1	[1 - 1]	0.999	[0.986 - 1]
		AMR	1	[1 - 1]	1	[1 - 1]	1	[1 - 1]	0.992	[0.941 - 1]
		EAS	1	[1 - 1]	1	[1 - 1]	1	[0.991 - 1]	0.990	[0.924 - 1]
		EUR	1	[1 - 1]	0.999	[0.992 - 1]	1	[1 - 1]	0.980	[0.904 - 1]
		SAS	1	[1 - 1]	1	[1 - 1]	1	[0.992 - 1]	0.979	[0.850 - 1]
	1260	AFR	1	[1 - 1]	1	[1 - 1]	1	[1 - 1]	0.999	[0.965 - 1]
		AMR	1	[1 - 1]	1	[1 - 1]	1	[0.989 - 1]	0.995	[0.918 - 1]
		EAS	1	[1 - 1]	1	[0.992 - 1]	1	[0.992 - 1]	0.994	[0.954 - 1]
		EUR	1	[1 - 1]	1	[0.992 - 1]	1	[0.992 - 1]	0.987	[0.919 - 1]
		SAS	1	[1 - 1]	1	[0.992 - 1]	1	[0.992 - 1]	0.983	[0.882 - 1]
	1440	AFR	1	[1 - 1]	1	[1 - 1]	1	[1 - 1]	0.999	[0.994 - 1]
		AMR	1	[1 - 1]	1	[1 - 1]	1	[0.989 - 1]	0.996	[0.957 - 1]
		EAS	1	[1 - 1]	1	[0.992 - 1]	1	[1 - 1]	0.995	[0.969 - 1]
		EUR	1	[1 - 1]	1	[1 - 1]	1	[1 - 1]	0.992	[0.969 - 1]
		SAS	1	[1 - 1]	1	[0.991 - 1]	1	[0.992 - 1]	0.984	[0.944 - 1]
	1620	AFR	1	[1 - 1]	1	[0.995 - 1]	1	[1 - 1]	0.999	[0.988 - 1]
		AMR	1	[1 - 1]	1	[1 - 1]	1	[1 - 1]	0.996	[0.921 - 1]
		EAS	1	[1 - 1]	1	[1 - 1]	1	[0.992 - 1]	0.995	[0.951 - 1]
		EUR	1	[1 - 1]	1	[0.993 - 1]	1	[1 - 1]	0.988	[0.831 - 1]
		SAS	1	[1 - 1]	1	[1 - 1]	1	[0.99 - 1]	0.986	[0.874 - 1]
	1800	AFR	1	[1 - 1]	1	[1 - 1]	1	[1 - 1]	1	[0.993 - 1]
		AMR	1	[1 - 1]	1	[1 - 1]	1	[1 - 1]	0.997	[0.953 - 1]
		EAS	1	[1 - 1]	1	[1 - 1]	1	[1 - 1]	0.996	[0.955 - 1]

		EUR	1	[1 - 1]	1	[1 - 1]	1	[0.992 - 1]	0.988	[0.912 - 1]
		SAS	1	[1 - 1]	1	[0.992 - 1]	1	[1 - 1]	0.985	[0.850 - 1]
MAF≥10%, Distance≤0.125Mb	180	AFR	0.998	[0.976 - 1]	0.946	[0.884 - 0.994]	0.956	[0.915 - 0.993]	0.566	[0.227 - 0.793]
		AMR	0.998	[0.957 - 1]	0.965	[0.876 - 1]	0.967	[0.870 - 1]	0.422	[0.034 - 0.764]
		EAS	0.992	[0.922 - 1]	0.938	[0.828 - 1]	0.948	[0.836 - 1]	0.378	[0.062 - 0.771]
		EUR	0.996	[0.951 - 1]	0.942	[0.817 - 1]	0.949	[0.852 - 1]	0.341	[0.048 - 0.701]
		SAS	0.993	[0.953 - 1]	0.929	[0.832 - 0.992]	0.942	[0.800 - 1]	0.324	[0.064 - 0.652]
	360	AFR	1	[1 - 1]	0.999	[0.993 - 1]	0.999	[0.989 - 1]	0.962	[0.805 - 1]
		AMR	1	[1 - 1]	0.999	[0.976 - 1]	0.998	[0.986 - 1]	0.882	[0.453 - 1]
		EAS	1	[1 - 1]	0.997	[0.978 - 1]	0.998	[0.984 - 1]	0.846	[0.336 - 0.984]
		EUR	1	[1 - 1]	0.996	[0.983 - 1]	0.997	[0.974 - 1]	0.819	[0.315 - 0.968]
		SAS	1	[1 - 1]	0.995	[0.971 - 1]	0.997	[0.969 - 1]	0.782	[0.306 - 0.951]
	540	AFR	1	[1 - 1]	1	[1 - 1]	1	[1 - 1]	0.99	[0.884 - 1]
		AMR	1	[1 - 1]	1	[0.988 - 1]	1	[0.989 - 1]	0.956	[0.552 - 1]
		EAS	1	[1 - 1]	0.999	[0.984 - 1]	0.999	[0.977 - 1]	0.948	[0.511 - 1]
		EUR	1	[1 - 1]	0.999	[0.992 - 1]	1	[0.991 - 1]	0.918	[0.387 - 1]
		SAS	1	[1 - 1]	0.999	[0.990 - 1]	0.999	[0.981 - 1]	0.904	[0.446 - 0.992]
	720	AFR	1	[1 - 1]	1	[1 - 1]	1	[1 - 1]	0.997	[0.953 - 1]
		AMR	1	[1 - 1]	1	[0.988 - 1]	1	[0.989 - 1]	0.982	[0.770 - 1]
		EAS	1	[1 - 1]	1	[0.991 - 1]	1	[0.991 - 1]	0.981	[0.756 - 1]
		EUR	1	[1 - 1]	0.999	[0.993 - 1]	1	[0.993 - 1]	0.965	[0.645 - 1]
		SAS	1	[1 - 1]	1	[0.991 - 1]	1	[0.990 - 1]	0.955	[0.750 - 1]
	900	AFR	1	[1 - 1]	1	[1 - 1]	1	[1 - 1]	0.999	[0.984 - 1]
		AMR	1	[1 - 1]	1	[1 - 1]	1	[1 - 1]	0.993	[0.897 - 1]
		EAS	1	[1 - 1]	1	[0.992 - 1]	1	[0.992 - 1]	0.991	[0.901 - 1]
		EUR	1	[1 - 1]	1	[0.992 - 1]	1	[1 - 1]	0.983	[0.815 - 1]

		SAS	1	[1 - 1]	1	[0.991 - 1]	1	[0.992 - 1]	0.977	[0.808 - 1]
	1080	AFR	1	[1 - 1]	1	[1 - 1]	1	[1 - 1]	0.999	[0.988 - 1]
		AMR	1	[1 - 1]	1	[1 - 1]	1	[0.989 - 1]	0.995	[0.957 - 1]
		EAS	1	[1 - 1]	1	[1 - 1]	1	[0.992 - 1]	0.992	[0.939 - 1]
		EUR	1	[1 - 1]	1	[1 - 1]	1	[1 - 1]	0.985	[0.926 - 1]
		SAS	1	[1 - 1]	1	[0.991 - 1]	1	[0.992 - 1]	0.978	[0.843 - 1]
	1260	AFR	1	[1 - 1]	1	[1 - 1]	1	[1 - 1]	0.999	[0.986 - 1]
		AMR	1	[1 - 1]	1	[1 - 1]	1	[1 - 1]	0.991	[0.880 - 1]
		EAS	1	[1 - 1]	1	[0.992 - 1]	1	[0.992 - 1]	0.992	[0.904 - 1]
		EUR	1	[1 - 1]	1	[0.992 - 1]	1	[1 - 1]	0.983	[0.863 - 1]
		SAS	1	[1 - 1]	1	[0.991 - 1]	1	[0.991 - 1]	0.978	[0.803 - 1]
	1440	AFR	1	[1 - 1]	1	[1 - 1]	1	[1 - 1]	0.999	[0.984 - 1]
		AMR	1	[1 - 1]	1	[1 - 1]	1	[1 - 1]	0.994	[0.940 - 1]
		EAS	1	[1 - 1]	1	[0.992 - 1]	1	[1 - 1]	0.992	[0.896 - 1]
		EUR	1	[1 - 1]	1	[1 - 1]	1	[0.991 - 1]	0.986	[0.906 - 1]
		SAS	1	[1 - 1]	1	[0.992 - 1]	1	[1 - 1]	0.981	[0.832 - 1]
	1620	AFR	1	[1 - 1]	1	[1 - 1]	1	[1 - 1]	0.999	[0.976 - 1]
		AMR	1	[1 - 1]	1	[1 - 1]	1	[1 - 1]	0.998	[0.976 - 1]
		EAS	1	[1 - 1]	1	[1 - 1]	1	[0.992 - 1]	0.995	[0.970 - 1]
		EUR	1	[1 - 1]	1	[0.993 - 1]	1	[0.992 - 1]	0.992	[0.921 - 1]
		SAS	1	[1 - 1]	1	[1 - 1]	1	[1 - 1]	0.988	[0.927 - 1]
	1800	AFR	1	[1 - 1]	1	[1 - 1]	1	[1 - 1]	0.999	[0.994 - 1]
		AMR	1	[1 - 1]	1	[1 - 1]	1	[1 - 1]	0.996	[0.941 - 1]
		EAS	1	[1 - 1]	1	[1 - 1]	1	[0.991 - 1]	0.996	[0.977 - 1]
		EUR	1	[1 - 1]	1	[1 - 1]	1	[0.992 - 1]	0.992	[0.945 - 1]
		SAS	1	[1 - 1]	1	[0.992 - 1]	1	[0.990 - 1]	0.988	[0.919 - 1]

Pop-MAF>0%, Distance≤0.0625Mb	180	AFR	1	[0.984 - 1]	0.965	[0.919 - 1]	0.973	[0.939 - 1]	0.622	[0.323 - 0.842]
		AMR	1	[0.988 - 1]	0.976	[0.929 - 1]	0.978	[0.921 - 1]	0.521	[0.045 - 0.929]
		EAS	0.999	[0.983 - 1]	0.972	[0.902 - 1]	0.978	[0.914 - 1]	0.565	[0.160 - 0.949]
		EUR	0.999	[0.979 - 1]	0.962	[0.887 - 1]	0.972	[0.919 - 1]	0.466	[0.063 - 0.875]
		SAS	0.999	[0.984 - 1]	0.960	[0.893 - 1]	0.969	[0.912 - 1]	0.486	[0.062 - 0.864]
	360	AFR	1	[1 - 1]	1	[0.994 - 1]	1	[0.994 - 1]	0.982	[0.896 - 1]
		AMR	1	[1 - 1]	0.998	[0.976 - 1]	0.999	[0.976 - 1]	0.937	[0.682 - 1]
		EAS	1	[1 - 1]	0.999	[0.984 - 1]	0.999	[0.991 - 1]	0.946	[0.727 - 1]
		EUR	1	[1 - 1]	0.997	[0.984 - 1]	0.998	[0.984 - 1]	0.892	[0.646 - 0.986]
		SAS	1	[1 - 1]	0.996	[0.982 - 1]	0.997	[0.981 - 1]	0.885	[0.616 - 0.984]
	540	AFR	1	[1 - 1]	1	[0.994 - 1]	1	[0.995 - 1]	0.993	[0.951 - 1]
		AMR	1	[1 - 1]	1	[0.986 - 1]	1	[0.986 - 1]	0.976	[0.886 - 1]
		EAS	1	[1 - 1]	1	[0.991 - 1]	1	[0.991 - 1]	0.976	[0.847 - 1]
		EUR	1	[1 - 1]	0.999	[0.992 - 1]	1	[0.992 - 1]	0.952	[0.806 - 1]
		SAS	1	[1 - 1]	0.999	[0.991 - 1]	0.999	[0.990 - 1]	0.943	[0.750 - 1]
	720	AFR	1	[1 - 1]	1	[1 - 1]	1	[1 - 1]	0.996	[0.936 - 1]
		AMR	1	[1 - 1]	1	[0.986 - 1]	1	[1 - 1]	0.983	[0.919 - 1]
		EAS	1	[1 - 1]	1	[0.992 - 1]	1	[0.991 - 1]	0.980	[0.855 - 1]
		EUR	1	[1 - 1]	1	[0.991 - 1]	1	[0.992 - 1]	0.963	[0.677 - 1]
		SAS	1	[1 - 1]	0.999	[0.991 - 1]	0.999	[0.991 - 1]	0.952	[0.784 - 1]
	900	AFR	1	[1 - 1]	1	[1 - 1]	1	[1 - 1]	0.997	[0.979 - 1]
		AMR	1	[1 - 1]	1	[0.986 - 1]	1	[0.989 - 1]	0.989	[0.921 - 1]
		EAS	1	[1 - 1]	1	[0.993 - 1]	1	[0.992 - 1]	0.990	[0.939 - 1]
		EUR	1	[1 - 1]	1	[0.993 - 1]	1	[0.992 - 1]	0.980	[0.875 - 1]
		SAS	1	[1 - 1]	1	[0.991 - 1]	1	[0.991 - 1]	0.971	[0.843 - 1]
	1080	AFR	1	[1 - 1]	1	[1 - 1]	1	[1 - 1]	0.997	[0.979 - 1]

		AMR	1	[1 - 1]	1	[0.986 - 1]	1	[1 - 1]	0.992	[0.943 - 1]
		EAS	1	[1 - 1]	1	[1 - 1]	1	[0.992 - 1]	0.992	[0.946 - 1]
		EUR	1	[1 - 1]	1	[0.992 - 1]	1	[1 - 1]	0.985	[0.871 - 1]
		SAS	1	[1 - 1]	1	[0.990 - 1]	0.999	[0.990 - 1]	0.977	[0.906 - 1]
	1260	AFR	1	[1 - 1]	1	[1 - 1]	1	[1 - 1]	0.998	[0.983 - 1]
		AMR	1	[1 - 1]	1	[1 - 1]	1	[1 - 1]	0.992	[0.943 - 1]
		EAS	1	[1 - 1]	1	[1 - 1]	1	[1 - 1]	0.993	[0.941 - 1]
		EUR	1	[1 - 1]	1	[0.991 - 1]	1	[0.992 - 1]	0.983	[0.914 - 1]
		SAS	1	[1 - 1]	1	[0.991 - 1]	1	[0.990 - 1]	0.976	[0.898 - 1]
	1440	AFR	1	[1 - 1]	1	[1 - 1]	1	[1 - 1]	0.998	[0.979 - 1]
		AMR	1	[1 - 1]	1	[0.986 - 1]	1	[1 - 1]	0.993	[0.929 - 1]
		EAS	1	[1 - 1]	1	[1 - 1]	1	[1 - 1]	0.993	[0.932 - 1]
		EUR	1	[1 - 1]	1	[0.992 - 1]	1	[1 - 1]	0.985	[0.889 - 1]
		SAS	1	[1 - 1]	1	[0.991 - 1]	0.999	[0.991 - 1]	0.974	[0.874 - 1]
	1620	AFR	1	[1 - 1]	1	[1 - 1]	1	[1 - 1]	0.998	[0.981 - 1]
		AMR	1	[1 - 1]	1	[0.986 - 1]	1	[1 - 1]	0.993	[0.918 - 1]
		EAS	1	[1 - 1]	1	[1 - 1]	1	[1 - 1]	0.992	[0.938 - 1]
		EUR	1	[1 - 1]	1	[0.992 - 1]	1	[0.992 - 1]	0.986	[0.898 - 1]
		SAS	1	[1 - 1]	1	[0.991 - 1]	1	[0.991 - 1]	0.978	[0.882 - 1]
	1800	AFR	1	[1 - 1]	1	[1 - 1]	1	[1 - 1]	0.998	[0.979 - 1]
		AMR	1	[1 - 1]	1	[1 - 1]	1	[1 - 1]	0.994	[0.953 - 1]
		EAS	1	[1 - 1]	1	[1 - 1]	1	[1 - 1]	0.993	[0.946 - 1]
		EUR	1	[1 - 1]	1	[0.991 - 1]	1	[1 - 1]	0.989	[0.923 - 1]
		SAS	1	[1 - 1]	1	[0.990 - 1]	0.999	[0.990 - 1]	0.977	[0.882 - 1]
Pop-MAF>0%, Distance≤0.125Mb	180	AFR	0.971	[0.855 - 1]	0.856	[0.727 - 0.968]	0.879	[0.767 - 0.975]	0.337	[0.050 - 0.717]
		AMR	0.990	[0.930 - 1]	0.927	[0.802 - 1]	0.937	[0.843 - 0.989]	0.264	[0.022 - 0.706]

		EAS	0.982	[0.889 - 1]	0.890	[0.748 - 0.984]	0.910	[0.785 - 1]	0.246	[0.008 - 0.63]
		EUR	0.989	[0.948 - 1]	0.901	[0.784 - 0.975]	0.917	[0.828 - 0.978]	0.218	[0.008 - 0.528]
		SAS	0.987	[0.901 - 1]	0.887	[0.757 - 0.973]	0.905	[0.793 - 0.976]	0.206	[0 - 0.606]
	360	AFR	1	[1 - 1]	0.997	[0.977 - 1]	0.998	[0.988 - 1]	0.928	[0.690 - 0.995]
		AMR	1	[1 - 1]	0.996	[0.965 - 1]	0.997	[0.966 - 1]	0.841	[0.322 - 1]
		EAS	1	[1 - 1]	0.992	[0.959 - 1]	0.993	[0.959 - 1]	0.812	[0.344 - 0.992]
		EUR	1	[1 - 1]	0.99	[0.952 - 1]	0.994	[0.966 - 1]	0.754	[0.260 - 0.941]
		SAS	1	[1 - 1]	0.989	[0.948 - 1]	0.992	[0.953 - 1]	0.749	[0.299 - 0.951]
	540	AFR	1	[1 - 1]	1	[0.994 - 1]	1	[0.995 - 1]	0.986	[0.913 - 1]
		AMR	1	[1 - 1]	1	[0.986 - 1]	0.999	[0.988 - 1]	0.960	[0.711 - 1]
		EAS	1	[1 - 1]	0.999	[0.983 - 1]	0.999	[0.981 - 1]	0.936	[0.623 - 1]
		EUR	1	[1 - 1]	0.999	[0.984 - 1]	0.999	[0.991 - 1]	0.915	[0.605 - 0.993]
		SAS	1	[1 - 1]	0.999	[0.984 - 1]	0.999	[0.990 - 1]	0.911	[0.560 - 1]
	720	AFR	1	[1 - 1]	1	[0.995 - 1]	1	[0.995 - 1]	0.994	[0.942 - 1]
		AMR	1	[1 - 1]	1	[0.988 - 1]	1	[1 - 1]	0.979	[0.816 - 1]
		EAS	1	[1 - 1]	0.999	[0.985 - 1]	1	[0.992 - 1]	0.974	[0.817 - 1]
		EUR	1	[1 - 1]	0.999	[0.992 - 1]	1	[0.991 - 1]	0.953	[0.718 - 1]
		SAS	1	[1 - 1]	0.999	[0.984 - 1]	0.999	[0.990 - 1]	0.943	[0.732 - 1]
	900	AFR	1	[1 - 1]	1	[1 - 1]	1	[1 - 1]	0.997	[0.942 - 1]
		AMR	1	[1 - 1]	1	[0.988 - 1]	1	[0.989 - 1]	0.987	[0.793 - 1]
		EAS	1	[1 - 1]	1	[0.992 - 1]	1	[0.992 - 1]	0.985	[0.786 - 1]
		EUR	1	[1 - 1]	1	[0.991 - 1]	1	[0.991 - 1]	0.975	[0.685 - 1]
		SAS	1	[1 - 1]	1	[0.992 - 1]	1	[1 - 1]	0.970	[0.714 - 1]
	1080	AFR	1	[1 - 1]	1	[1 - 1]	1	[1 - 1]	0.997	[0.977 - 1]
		AMR	1	[1 - 1]	1	[1 - 1]	1	[1 - 1]	0.989	[0.931 - 1]
		EAS	1	[1 - 1]	1	[0.992 - 1]	1	[0.992 - 1]	0.990	[0.924 - 1]

		EUR	1	[1 - 1]	1	[0.992 - 1]	1	[0.991 - 1]	0.979	[0.863 - 1]
		SAS	1	[1 - 1]	1	[1 - 1]	1	[0.992 - 1]	0.975	[0.864 - 1]
	1260	AFR	1	[1 - 1]	1	[1 - 1]	1	[1 - 1]	0.999	[0.988 - 1]
		AMR	1	[1 - 1]	1	[0.986 - 1]	1	[1 - 1]	0.993	[0.921 - 1]
		EAS	1	[1 - 1]	1	[1 - 1]	1	[0.991 - 1]	0.990	[0.908 - 1]
		EUR	1	[1 - 1]	1	[0.992 - 1]	1	[0.991 - 1]	0.983	[0.839 - 1]
		SAS	1	[1 - 1]	1	[0.990 - 1]	1	[1 - 1]	0.977	[0.883 - 1]
	1440	AFR	1	[1 - 1]	1	[1 - 1]	1	[1 - 1]	0.999	[0.988 - 1]
		AMR	1	[1 - 1]	1	[1 - 1]	1	[1 - 1]	0.997	[0.965 - 1]
		EAS	1	[1 - 1]	1	[0.992 - 1]	1	[0.992 - 1]	0.995	[0.958 - 1]
		EUR	1	[1 - 1]	1	[0.993 - 1]	1	[0.993 - 1]	0.989	[0.956 - 1]
		SAS	1	[1 - 1]	1	[0.991 - 1]	1	[0.991 - 1]	0.985	[0.917 - 1]
	1620	AFR	1	[1 - 1]	1	[1 - 1]	1	[1 - 1]	0.999	[0.994 - 1]
		AMR	1	[1 - 1]	1	[1 - 1]	1	[0.989 - 1]	0.997	[0.976 - 1]
		EAS	1	[1 - 1]	1	[1 - 1]	1	[1 - 1]	0.995	[0.972 - 1]
		EUR	1	[1 - 1]	1	[0.993 - 1]	1	[0.993 - 1]	0.991	[0.960 - 1]
		SAS	1	[1 - 1]	1	[1 - 1]	1	[0.991 - 1]	0.987	[0.919 - 1]
	1800	AFR	1	[1 - 1]	1	[1 - 1]	1	[1 - 1]	0.999	[0.988 - 1]
		AMR	1	[1 - 1]	1	[1 - 1]	1	[0.989 - 1]	0.997	[0.953 - 1]
		EAS	1	[1 - 1]	1	[1 - 1]	1	[1 - 1]	0.995	[0.939 - 1]
		EUR	1	[1 - 1]	1	[0.993 - 1]	1	[1 - 1]	0.990	[0.919 - 1]
		SAS	1	[1 - 1]	1	[0.991 - 1]	1	[0.991 - 1]	0.988	[0.911 - 1]
Pop-MAF≥1%, Distance≤0.0625Mb	180	AFR	1	[0.987 - 1]	0.973	[0.905 - 1]	0.978	[0.917 - 1]	0.687	[0.238 - 0.890]
		AMR	1	[0.988 - 1]	0.986	[0.929 - 1]	0.989	[0.955 - 1]	0.575	[0.058 - 0.860]
		EAS	1	[0.983 - 1]	0.983	[0.938 - 1]	0.986	[0.953 - 1]	0.609	[0.109 - 0.914]
		EUR	1	[0.985 - 1]	0.974	[0.929 - 1]	0.982	[0.933 - 1]	0.514	[0.069 - 0.813]

		SAS	0.999	[0.984 - 1]	0.977	[0.921 - 1]	0.981	[0.921 - 1]	0.525	[0.009 - 0.820]
	360	AFR	1	[1 - 1]	0.999	[0.990 - 1]	1	[0.995 - 1]	0.978	[0.643 - 1]
		AMR	1	[1 - 1]	0.999	[0.977 - 1]	1	[0.988 - 1]	0.934	[0.081 - 1]
		EAS	1	[1 - 1]	0.999	[0.991 - 1]	1	[0.992 - 1]	0.938	[0.180 - 1]
		EUR	1	[1 - 1]	0.998	[0.983 - 1]	0.999	[0.991 - 1]	0.895	[0.069 - 0.993]
		SAS	1	[1 - 1]	0.998	[0.976 - 1]	0.999	[0.984 - 1]	0.893	[0.108 - 0.992]
	540	AFR	1	[1 - 1]	1	[1 - 1]	1	[1 - 1]	0.992	[0.890 - 1]
		AMR	1	[1 - 1]	0.999	[0.986 - 1]	1	[0.989 - 1]	0.978	[0.759 - 1]
		EAS	1	[1 - 1]	1	[0.992 - 1]	1	[0.992 - 1]	0.981	[0.794 - 1]
		EUR	1	[1 - 1]	1	[0.992 - 1]	1	[0.991 - 1]	0.96	[0.613 - 1]
		SAS	1	[1 - 1]	1	[0.991 - 1]	0.999	[0.990and - 1]	0.948	[0.723 - 1]
	720	AFR	1	[1 - 1]	1	[1 - 1]	1	[1 - 1]	0.996	[0.969 - 1]
		AMR	1	[1 - 1]	1	[0.986 - 1]	1	[1 - 1]	0.983	[0.882 - 1]
		EAS	1	[1 - 1]	1	[0.992 - 1]	1	[0.992 - 1]	0.985	[0.917 - 1]
		EUR	1	[1 - 1]	1	[0.992 - 1]	1	[0.993 - 1]	0.968	[0.803 - 1]
		SAS	1	[1 - 1]	1	[0.991 - 1]	1	[0.992 - 1]	0.959	[0.780 - 1]
	900	AFR	1	[1 - 1]	1	[1 - 1]	1	[1 - 1]	0.998	[0.983 - 1]
		AMR	1	[1 - 1]	1	[0.986 - 1]	1	[1 - 1]	0.988	[0.906 - 1]
		EAS	1	[1 - 1]	1	[1 - 1]	1	[1 - 1]	0.991	[0.947 - 1]
		EUR	1	[1 - 1]	1	[1 - 1]	1	[1 - 1]	0.983	[0.915 - 1]
		SAS	1	[1 - 1]	0.999	[0.990 - 1]	0.999	[0.990 - 1]	0.968	[0.843 - 1]
	1080	AFR	1	[1 - 1]	1	[1 - 1]	1	[1 - 1]	0.999	[0.989 - 1]
		AMR	1	[1 - 1]	1	[1 - 1]	1	[1 - 1]	0.993	[0.953 - 1]
		EAS	1	[1 - 1]	1	[1 - 1]	1	[0.992 - 1]	0.991	[0.947 - 1]
		EUR	1	[1 - 1]	1	[1 - 1]	1	[1 - 1]	0.987	[0.923 - 1]
		SAS	1	[1 - 1]	0.999	[0.984 - 1]	0.999	[0.991 - 1]	0.976	[0.906 - 1]

	1260	AFR	1	[1 - 1]	1	[1 - 1]	1	[1 - 1]	0.998	[0.977 - 1]
		AMR	1	[1 - 1]	1	[1 - 1]	1	[1 - 1]	0.992	[0.941 - 1]
		EAS	1	[1 - 1]	1	[1 - 1]	1	[1 - 1]	0.994	[0.945 - 1]
		EUR	1	[1 - 1]	1	[1 - 1]	1	[0.992 - 1]	0.988	[0.897 - 1]
		SAS	1	[1 - 1]	1	[0.992 - 1]	0.999	And [0.990 - 1]	0.977	[0.871 - 1]
	1440	AFR	1	[1 - 1]	1	[1 - 1]	1	[1 - 1]	0.998	[0.984 - 1]
		AMR	1	[1 - 1]	1	[0.986 - 1]	1	[1 - 1]	0.991	[0.894 - 1]
		EAS	1	[1 - 1]	1	[1 - 1]	1	[1 - 1]	0.993	[0.938 - 1]
		EUR	1	[1 - 1]	1	[1 - 1]	1	[0.992 - 1]	0.986	[0.932 - 1]
		SAS	1	[1 - 1]	1	[0.991 - 1]	1	[0.990 - 1]	0.977	[0.893 - 1]
	1620	AFR	1	[1 - 1]	1	[1 - 1]	1	[1 - 1]	0.998	[0.979 - 1]
		AMR	1	[1 - 1]	1	[1 - 1]	1	[1 - 1]	0.993	[0.918 - 1]
		EAS	1	[1 - 1]	1	[0.992 - 1]	1	[1 - 1]	0.994	[0.939 - 1]
		EUR	1	[1 - 1]	1	[1 - 1]	1	[0.992 - 1]	0.987	[0.911 - 1]
		SAS	1	[1 - 1]	1	[0.992 - 1]	0.999	[0.991 - 1]	0.979	[0.874 - 1]
	1800	AFR	1	[1 - 1]	1	[1 - 1]	1	[0.995 - 1]	0.998	[0.983 - 1]
		AMR	1	[1 - 1]	1	[0.989 - 1]	1	[0.988 - 1]	0.993	[0.906 - 1]
		EAS	1	[1 - 1]	1	[1 - 1]	1	[1 - 1]	0.995	[0.955 - 1]
		EUR	1	[1 - 1]	1	[0.993 - 1]	1	[1 - 1]	0.989	[0.919 - 1]
		SAS	1	[1 - 1]	1	[0.991 - 1]	0.999	[0.990 - 1]	0.979	[0.874 - 1]
Pop-MAF≥5%, Distance≤0.125Mb	180	AFR	0.998	[0.953 - 1]	0.946	[0.872 - 1]	0.957	[0.895 - 1]	0.577	[0.262 - 0.848]
		AMR	0.999	[0.976 - 1]	0.966	[0.899 - 1]	0.971	[0.884 - 1]	0.448	[0.094 - 0.787]
		EAS	0.997	[0.958 - 1]	0.950	[0.859 - 1]	0.957	[0.866 - 1]	0.426	[0.080 - 0.782]
		EUR	0.998	[0.976 - 1]	0.950	[0.86 - 0.992]	0.964	[0.897 - 1]	0.392	[0.113 - 0.731]
		SAS	0.998	[0.971 - 1]	0.946	[0.874 - 1]	0.951	[0.864 - 1]	0.355	[0.055 - 0.685]
	360	AFR	1	[1 - 1]	0.999	[0.993 - 1]	1	[0.994 - 1]	0.969	[0.767 - 1]

		AMR	1	[1 - 1]	0.999	[0.988 - 1]	0.998	[0.967 - 1]	0.910	[0.506 - 0.989]
		EAS	1	[1 - 1]	0.998	[0.985 - 1]	0.999	[0.991 - 1]	0.890	[0.427 - 0.992]
		EUR	1	[1 - 1]	0.997	[0.978 - 1]	0.998	[0.984 - 1]	0.861	[0.363 - 0.985]
		SAS	1	[1 - 1]	0.998	[0.983 - 1]	0.998	[0.984 - 1]	0.853	[0.393 - 0.991]
	540	AFR	1	[1 - 1]	1	[1 - 1]	1	[1 - 1]	0.995	[0.924 - 1]
		AMR	1	[1 - 1]	1	[0.986 - 1]	1	[0.988 - 1]	0.976	[0.793 - 1]
		EAS	1	[1 - 1]	1	[0.992 - 1]	1	[0.991 - 1]	0.973	[0.809 - 1]
		EUR	1	[1 - 1]	0.998	[0.984 - 1]	1	[0.992 - 1]	0.957	[0.758 - 1]
		SAS	1	[1 - 1]	0.999	[0.990 - 1]	1	[0.990 - 1]	0.943	[0.776 - 1]
	720	AFR	1	[1 - 1]	1	[1 - 1]	1	[1 - 1]	0.998	[0.971 - 1]
		AMR	1	[1 - 1]	1	[0.988 - 1]	1	[1 - 1]	0.988	[0.899 - 1]
		EAS	1	[1 - 1]	1	[0.992 - 1]	1	[0.992 - 1]	0.987	[0.878 - 1]
		EUR	1	[1 - 1]	1	[0.993 - 1]	1	[0.992 - 1]	0.978	[0.831 - 1]
		SAS	1	[1 - 1]	1	[0.990 - 1]	1	[0.990 - 1]	0.968	[0.874 - 1]
	900	AFR	1	[1 - 1]	1	[1 - 1]	1	[1 - 1]	0.999	[0.988 - 1]
		AMR	1	[1 - 1]	1	[1 - 1]	1	[1 - 1]	0.993	[0.935 - 1]
		EAS	1	[1 - 1]	1	[1 - 1]	1	[0.992 - 1]	0.992	[0.955 - 1]
		EUR	1	[1 - 1]	1	[0.992 - 1]	1	[0.993 - 1]	0.985	[0.923 - 1]
		SAS	1	[1 - 1]	1	[0.991 - 1]	1	[0.992 - 1]	0.98	[0.904 - 1]
	1080	AFR	1	[1 - 1]	1	[1 - 1]	1	[1 - 1]	0.999	[0.984 - 1]
		AMR	1	[1 - 1]	1	[1 - 1]	1	[1 - 1]	0.993	[0.871 - 1]
		EAS	1	[1 - 1]	1	[0.992 - 1]	1	[1 - 1]	0.989	[0.894 - 1]
		EUR	1	[1 - 1]	1	[0.993 - 1]	1	[0.992 - 1]	0.986	[0.879 - 1]
		SAS	1	[1 - 1]	1	[0.990 - 1]	1	[0.991 - 1]	0.975	[0.772 - 1]
	1260	AFR	1	[1 - 1]	1	[1 - 1]	1	[1 - 1]	0.999	[0.979 - 1]
		AMR	1	[1 - 1]	1	[1 - 1]	1	[1 - 1]	0.995	[0.952 - 1]

		EAS	1	[1 - 1]	1	[1 - 1]	1	[1 - 1]	0.995	[0.962 - 1]
		EUR	1	[1 - 1]	1	[0.992 - 1]	1	[0.991 - 1]	0.989	[0.949 - 1]
		SAS	1	[1 - 1]	1	[0.992 - 1]	1	[0.992 - 1]	0.983	[0.937 - 1]
		AFR	1	[1 - 1]	1	[1 - 1]	1	[1 - 1]	0.999	[0.988 - 1]
		AMR	1	[1 - 1]	1	[1 - 1]	1	[1 - 1]	0.996	[0.953 - 1]
	1440	EAS	1	[1 - 1]	1	[1 - 1]	1	[1 - 1]	0.995	[0.972 - 1]
		EUR	1	[1 - 1]	1	[1 - 1]	1	[0.992 - 1]	0.988	[0.945 - 1]
		SAS	1	[1 - 1]	1	[1 - 1]	1	[0.991 - 1]	0.984	[0.927 - 1]
		AFR	1	[1 - 1]	1	[1 - 1]	1	[1 - 1]	0.998	[0.984 - 1]
		AMR	1	[1 - 1]	1	[0.989 - 1]	1	[1 - 1]	0.997	[0.966 - 1]
	1620	EAS	1	[1 - 1]	1	[0.992 - 1]	1	[0.992 - 1]	0.997	[0.984 - 1]
		EUR	1	[1 - 1]	1	[0.991 - 1]	1	[0.992 - 1]	0.992	[0.935 - 1]
		SAS	1	[1 - 1]	1	[1 - 1]	1	[0.990 - 1]	0.987	[0.929 - 1]
		AFR	1	[1 - 1]	1	[1 - 1]	1	[1 - 1]	0.999	[0.989 - 1]
		AMR	1	[1 - 1]	1	[1 - 1]	1	[1 - 1]	0.997	[0.965 - 1]
	1800	EAS	1	[1 - 1]	1	[1 - 1]	1	[1 - 1]	0.995	[0.953 - 1]
		EUR	1	[1 - 1]	1	[0.993 - 1]	1	[0.991 - 1]	0.992	[0.957 - 1]
		SAS	1	[1 - 1]	1	[0.992 - 1]	1	[0.984 - 1]	0.983	[0.904 - 1]

Table S7. Record-matching accuracies in five super-populations for the six conditions that achieved comparable record-matching accuracy to the full data with 900 SNPs, as in Table 1C. To obtain record-matching accuracies for each super-population, we first obtained the match score matrices produced by experiments that considered all 2504 individuals, summarized in Figure 3 and Table S6. We annotated each individual by super-population, and for each super-population, we computed the record-matching accuracy in each of the four matching scenarios. Rows highlighted in light pink correspond to SNP sets that, for a given super-population, achieve median record-matching accuracies in the one-to-one, one-to-many with SNP query, one-to-many with STR query, and needle-in-haystack scenarios of 1, 1, 1, and ≥0.99. The table shows medians and ranges plotted in Figure S3.

		Ratio of posterior odds and prior odds																	
		10^0	10^1	10^2	10^3	10^4	10^5	10^6	10^7	10^8	10^9	10^{10}	10^{11}	10^{12}	10^{13}	10^{14}	10^{15}	10^{16}	10^{17}
		**Minimum match score**																	
		0	2.30	4.61	6.91	9.21	11.51	13.82	16.12	18.42	20.72	23.03	25.33	27.63	29.93	32.24	34.54	36.84	39.14
		**Fraction of true matches exceeding the minimum match score**																	
MAF≥5%, Distance≤0.125Mb	900	1	1	0.99	0.99	0.98	0.96	0.94	0.90	0.84	0.77	0.69	0.61	0.51	0.42	0.34	0.28	0.22	0.18
	1800	1	1	0.99	0.99	0.98	0.97	0.95	0.92	0.88	0.83	0.77	0.69	0.61	0.52	0.44	0.37	0.30	0.24
MAF≥10%, Distance≤0.125Mb	900	1	1	1	0.99	0.98	0.97	0.94	0.90	0.85	0.79	0.71	0.62	0.53	0.44	0.36	0.29	0.23	0.18
	1800	1	1	0.99	0.99	0.98	0.97	0.95	0.92	0.89	0.83	0.77	0.69	0.61	0.53	0.45	0.37	0.30	0.24
Pop-MAF>0%, Distance≤0.0625Mb	900	1	1	0.99	0.99	0.98	0.96	0.93	0.89	0.83	0.76	0.68	0.58	0.49	0.40	0.32	0.25	0.20	0.16
	1800	1	1	0.99	0.99	0.98	0.97	0.94	0.91	0.87	0.82	0.75	0.67	0.59	0.50	0.41	0.34	0.27	0.21
Pop-MAF>0%, Distance≤0.125Mb	900	1	1	0.99	0.99	0.98	0.96	0.93	0.89	0.84	0.77	0.69	0.60	0.51	0.42	0.34	0.27	0.21	0.17
	1800	1	1	0.99	0.99	0.98	0.97	0.95	0.93	0.89	0.84	0.78	0.70	0.62	0.53	0.45	0.37	0.30	0.24
Pop-MAF≥1%, Distance≤0.0625Mb	900	1	1	0.99	0.99	0.98	0.96	0.93	0.89	0.84	0.77	0.68	0.59	0.49	0.40	0.32	0.25	0.20	0.16
	1800	1	1	0.99	0.99	0.98	0.96	0.94	0.90	0.86	0.80	0.73	0.65	0.56	0.47	0.39	0.32	0.25	0.20
Pop-MAF≥5%, Distance≤0.125Mb	900	1	1	0.99	0.99	0.98	0.96	0.94	0.90	0.85	0.79	0.71	0.62	0.53	0.44	0.36	0.29	0.23	0.18
	1800	1	1	0.99	0.99	0.98	0.97	0.95	0.92	0.88	0.83	0.77	0.70	0.62	0.53	0.45	0.37	0.30	0.24
All SNPs around 18 STRs	192,672	0.99	0.99	0.99	0.98	0.97	0.96	0.94	0.91	0.88	0.85	0.80	0.75	0.69	0.63	0.57	0.50	0.43	0.36
All SNPs around 15 STRs	161,968	0.98	0.97	0.96	0.94	0.92	0.89	0.85	0.80	0.74	0.68	0.61	0.53	0.44	0.36	0.29	0.22	0.16	0.12
All SNPs around 15 STRs [11]	161,968	0.99	0.99	0.98	0.96	0.95	0.93	0.89	0.85	0.81	0.75	0.67	0.58	0.47	0.37	0.27	0.19	0.12	0.09

Table S8. The fraction of true matches with match score exceeding the minimum threshold for achieving a desired ratio of posterior and prior odds. The numerical values are taken from analyses with 900 and 1800 SNPs that appear in Table 1C and Figure 3. For comparison, corresponding values for the full set of 192,672 SNPs as shown in Figure 1, and for the 161,968 SNPs with only the 15 STR loci considered by [11], are also included. The table is comparable to Table 2 of [11]; the last line shows the values from corresponding analyses in Table 2 of [11].
